# X-chromosomal STR based genetic polymorphisms and demographic history of Sri Lankan ethnicities and their relationship with global populations

**DOI:** 10.1038/s41598-021-92314-9

**Published:** 2021-06-17

**Authors:** Nandika Perera, Gayani Galhena, Gaya Ranawaka

**Affiliations:** 1 Genetech Molecular Diagnostics, Colombo 08, Sri Lanka; 2 Faculty of Health Sciences, The Open University of Sri Lanka, Nawala, Sri Lanka; 3 Department of Zoology and Environment Sciences, University of Colombo, Colombo 03, Sri Lanka

**Keywords:** Evolution, Genetics, Molecular biology

## Abstract

A new 16 X-short tandem repeat (STR) multiplex PCR system has recently been developed for Sr Lankans, though its applicability in evolutionary genetics and forensic investigations has not been thoroughly assessed. In this study, 838 unrelated individuals covering all four major ethnic groups (Sinhalese, Sri Lankan Tamils, Indian Tamils and Moors) in Sri Lanka were successfully genotyped using this new multiplex system. The results indicated a high forensic efficiency for the tested loci in all four ethnicities confirming its suitability for forensic applications of Sri Lankans. Allele frequency distribution of Indian Tamils showed subtle but statistically significant differences from those of Sinhalese and Moors, in contrast to frequency distributions previously reported for autosomal STR alleles. This suggest a sex biased demographic history among Sri Lankans requiring a separate X-STR allele frequency database for Indian Tamils. Substantial differences observed in the patterns of LD among the four groups demand the use of a separate haplotype frequency databases for each individual ethnicity. When analysed together with other 14 world populations, all Sri Lankan ethnicities except Indian Tamils clustered closely with populations from Indian Bhil tribe, Bangladesh and Europe reflecting their shared Indo-Aryan ancestry.

## Introduction

Sri Lanka is an island country in South Asia, located in the Indian Ocean, close to India. Due to its strategic position at middle of the maritime silk route from China to Europe, it was well known to the outside world from ancient times as a trading hub. The diverse ethnicities that compose the 20 million population inhabiting the island as per the last population census^1^ have descended mainly from numerous groups of migrants who came to the island at various historical time periods. Their overpowering impact have confined the original inhabitants of the country to a few dry zone areas, forming a tribal group known as the Veddahs (aboriginals)^2^, represented by about 10,000 individuals^3^.

Today, the Sinhalese make the largest ethno-cultural group in Sri Lanka, having a population of 15.17 million (74.9% of the total population)^1^. The Sinhalese make a unique population in the world as the only ethnic group that speaks Sinhala, a branch of the Indo-European (Indo-Aryan) language family^4^. According to historical chronicles, the Bengali prince Vijaya and his seven hundred followers, who are descendants from the Indo-Aryans natives of the northern Indian subcontinent laid the foundation to the Sinhalese in 543 BCE^4^. They vanquished the aboriginal Veddas and converted Sri Lanka to a Sinhalese territory until the Dravidian rulers from South India invaded the northern part of the island in the fifth century AD^4^. Since then, there had been frequent migrations by South Indians into the country, which gave rise to the second largest ethnic group in Sri Lanka known as the “Ceylon Tamil” (2.27 million people representing 11.2%)^1^.

A third ethnic group was established in the country when Arab traders visiting the country for commercial purposes settled in Sri Lanka in 1000 AD, leading to intermarriages with the Sinhalese and the Sri Lankan Tamils^4^. The group now known as Moors comprise 9.2%^1^ (1.86 million) of the Sri Lankan population and maintain unique sociocultural features which are based largely on the Islamic faith. They speak a Dravidian language that contains large number of Arabic words that is generally referred to as ‘‘Arabic Tamil’’^5^. However, some scholars attribute the origin of Moors to South Indian traders, who later settled in Sri Lanka^5^. This view in part is based on the similarities shared by Sri Lankan Moors with the Tamil Muslims of Tamil Nadu. Indian Tamils make the 4th largest ethnic group in Sri Lanka and comprise the descendants from plantation workers brought to Sri Lanka from South India by the English rulers who colonized Sri Lanka in nineteenth century^4^. Comprising a relative minority of 0.84 million people (4.2% of the total population)^1^, Indian Tamils are chiefly confined to the central hills in Sri Lanka with a relatively low admixture with other ethnic groups due to socio cultural reasons associated with their more recent immigrant status.

In addition to these ethnicities, around 0.5% of the Sri Lankan population comprises other minor ethnic groups belonging to numerous descents. They include Malays (descendants from island of Java) Burghers (descendants of colonists from Portugal, Netherlands and UK) and other Chinese and African migrants who came to the island in the eighteenth and nineteenth centuries.

Because of the demographic history and geneflow, the genetic position occupied by each of these ethnic groups, both at local and global scales is not clear. The wide array of genetic markers currently available provide an opportunity for reliably elucidating their genetic affinities. However, depending on the type of genetic markers used, the ancestral information of populations deduced from the analyses could be quite different. On the other hand, each of these different approaches can complement each other with their characteristic genetic information. Although a few previous studies that had been conducted to understand the genetic substructure and underlying heterogeneity of Sri Lankan ethnicities using autosomal^6^, Y chromosomal^7^ and mitochondrial markers^8,9^, none has utilized those present on the X chromosome. X-chromosome markers, particularly short tandem repeats (STRs), with the advantageous features of both autosomal and uniparental biomarkers, play an important role in evolutionary studies^10,11^ as well as in forensic genetics^12^.They assist in the interpretation of complex kinship cases on its own or in conjunction with other marker like autosomal STRs. Analysis of X chromosome STRs (X-STRs) is specially advantageous in complex cases, where at least one female is involved, such as in a deficient paternity case of a female child, cases involving female siblings sharing a common biological father, questioned relationships between paternal grandmother-granddaughter or other distant female relatives^13^. Additionally, at some rare instances, they can also serve in forensic case work in which female traces are to be identified in male background contamination^13,14^. The use of clusters of tightly linked X-STRs forming highly informative haplotypes is particularly effective in such cases^14^. In addition, X-chromosome markers have a proven utility in tracing the sex biased demographic history among populations with complex admixture and geneflow patterns. Consequently, X-chromosome markers have gained significant importance in population and forensic genetic studies in the past 2 decades. However, the routine practice of molecular genetics in Sri Lanka presently comprise only of autosomal, Y-chromosomal and mitochondrial DNA analyses. The scope of X-chromosome markers is yet to be investigated for Sri Lankans.

**Figure 1:**
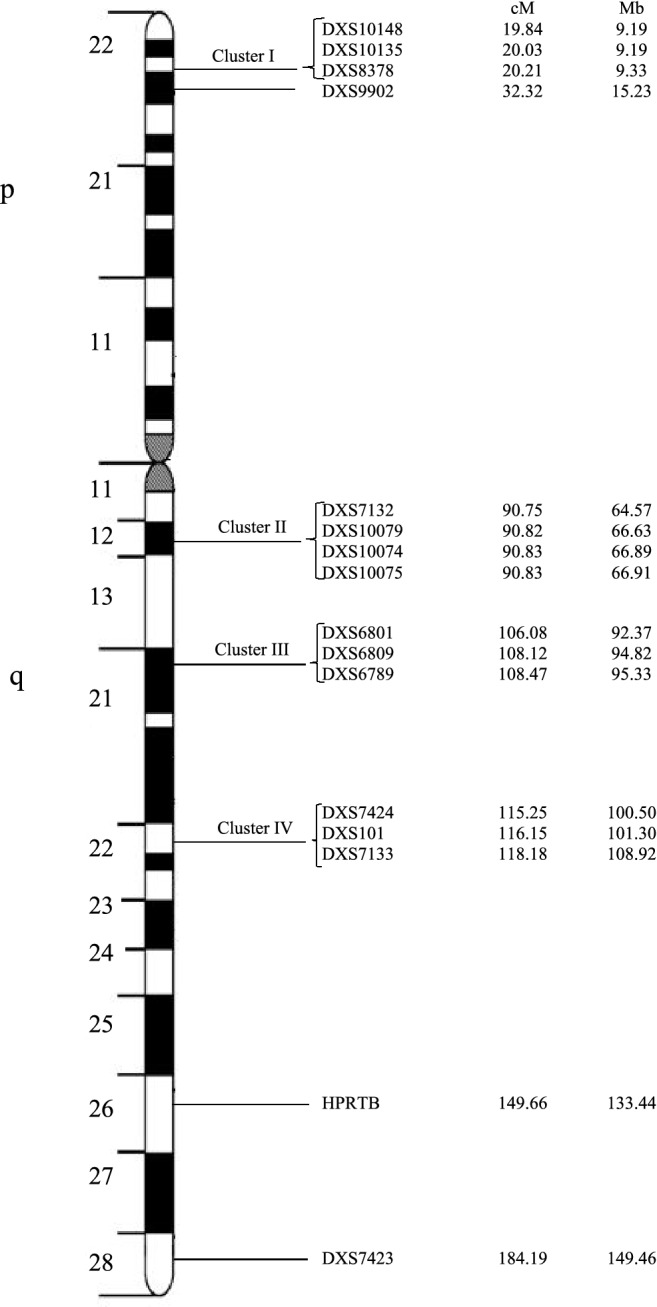
The ideogram of the X-chromosome describing the genetic positions of the 16 X-STR markers and their physical location. Distances from the p-telomere are shown in both cM and Mb. The order and approximate position of STRs are based upon ChrX-STR.org 2.0 database (http://www.chrx-str.org).

Recognizing this vital need exist in the field of molecular genetics in Sri Lanka, we recently developed a multiplex X-STR system with 16 X-STR markers (Fig. 1) distributed from 9.198 to 149.460 Mb of the X chromosome^15^ with the aim of incorporating X-STR analysis in to molecular forensic and evolutionary genetics investigations in the country. Thirteen of these STR markers are in four closely linked clusters (each spanning < 3 cM) that are likely to produce stable haplotypes (Cluster I; DXS10148-DXS10135-DXS8378 (Xp22), Cluster II; DXS7132-DXS10079-DXS10074-DXS10075 (Xq12), Cluster III: DXS6801-DXS6809-DXS6789 (Xq21), Cluster IV; DXS7424-DXS101-DXS7133 (Xq22)). Additional three unlinked markers were also included from both p (DXS9902 at Xp22) and q (HPRTB at Xq26 and DXS7423 at Xq28) arms to have a better coverage of the X chromosome. The assay was validated for the Sinhalese using 200 unrelated individuals of which 120 were males. In the present study, we extended the analysis to evaluate the forensic efficiency of this novel 16 X-STR assay to all four major ethnicities in Sri Lanka (Sinhalese, Sri Lankan Tamil, Indian Tamils and Moors) and constructed an allele and haplotype frequency database for Sri Lankans for forensic and kinship analysis purposes. Further, we have conducted a comprehensive analysis of the possible linkage disequilibrium (LD) among the selected X-STR markers. Here, we report for the first time, the X-STR based population genetic information of all four main ethnicities in Sri Lanka. In addition, Pairwise genetic distances based on Fst were also calculated between the Sri Lankan population and populations from South, South East and East Asia, Europe, Africa and Brazil based on the data extracted from literature to elucidate the genetic substructure between Sri Lankan and other global populations.

## Results and discussion

### Polymorphism

We typed 16 X-STR loci for 838 unrelated individuals of the Sri Lankan population covering the four major ethnicities (Supplementary Fig. S1). Complete DNA profiles were obtained for all male samples without any allele dropouts. Among the four ethnicities, the number of observed alleles varies from 4 to 25 across the 16 loci. When female samples were tested for the conformity to Hardy–Weinberg equilibrium, no significant deviations were observed for any of the tested loci after adjusting for multiple comparisons (Bonferroni corrected *P* = 0.0031) (Supplementary Table S1). The exact test of population differentiation did not detect significant differences in allele distribution among the male and female samples and hence the allele frequencies were combined for both sexes for further analysis. The allele frequencies of the four ethnicities are displayed in Supplementary Tables S2–S5.

The forensic parameters, expected heterozygosity (He), polymorphism information content (PIC), power of discrimination for males and females (PD_m_ and PD_f_), mean exclusion chance for trios and duos (MEC_trio_ and MEC _duo_) calculated for the 16 individual loci based on the allele frequency data (Supplementary Tables S6–S13) indicated high values in general for all markers. Expected heterozygosity (He) values were above 0.6 for all X-STR markers in all four ethnicities, except for DXS7423 in the two Tamil ethnicities. PIC values of 14 of the studied markers were above 0.6 for all ethnicities, among which, eight showed values above 0.7. Among all the tested loci, DXS10135 showed the highest value for all forensic parameters, suggesting it to be the most informative marker for Sri Lankans, while DXS7423 showed the lowest and the least informative. These results indicate that the 16 X-STR markers are polymorphic enough for both the forensic and kinship analysis applications.

### Population differentiation among Sri Lankan ethnicities

A locus-by-locus analysis of molecular variance (AMOVA) was carried out including all 16 loci, grouping the four ethnicities based on their linguistic origins (Table 1) to understand the extent of genetic differentiation among them. Out of the four populations, Sinhalese are known to have an Indo-Aryan origin, which is different from the Dravidian linguistic origin of the other three ethnicities. However, a significant variation was not detected among the two linguistic groups (Fct = − 0.00059; *P* > 0.05) though a subtle, but statistically significant variation was detected among populations within groups (Fsc = 0.0018; *P* = 0.0108). The global AMOVA results as a weighted average over loci showed that most of the variance in the samples is attributable to within-individual variation (97.97%) and between ethnic group variation is around 0.18%. To better understand this observed population structure, pairwise comparisons (pairwise Fst analysis) were carried out among all four ethnicities (Table 2). According to the Fst values obtained, Indian Tamils were shown to have a subtle but statistically significant genetic subdivision from Sinhalese (Fst = 0.0029; *P* = 0.0000) and Moors (Fst = 0.0038; *P* = 0.0000) while Sinhalese, Sri Lankan Tamils and Moors are highly panmictic (*P* > 0.05). Further, the two Tamil ethnicities were shown to share a common genetic background (*P* > 0.05).

**Table 1:** Analysis of molecular variance (AMOVA) among the four ethnicities based on linguistic groups.

Source of variation	Sum of squares	Variance components	Percentage of variation	Fixation indices	*P* value
Among groups	8.456	− 0.00363 Va	− 0.06	FCT: − 0.00059	0.80156
Among populations within groups	16.965	0.01091 Vb	0.18	FSC : 0.00178	0.01075
Among individuals within populations	3758.554	0.11718 Vc	1.91	FIS : 0.01913	0.00098
Within individuals	3641.500	6.00908 Vd	97.97	FIT: 0.02029	0.00000
Total	7425.474	6.13354

**Table 2:** Pairwise Fst and *P* values values for four ethnicities.

	Sinhala	SL Tamil	IND Tamil	Moors
Sinhala		0.18919 ± 0.0370	**0.00000 ± 0.0000**	0.36036 ± 0.0450
SL Tamil	0.00062		0.17117 ± 0.0394	0.08108 ± 0.0252
IND Tamil	0.00294	0.00082		**0.00000 ± 0.0000**
Moors	0.00033	0.00134	0.00375	

Below diagonal: pairwise Fst values, above diagonal: *P* values, significant *P* values are indicated in bold font.

**Figure 2:**
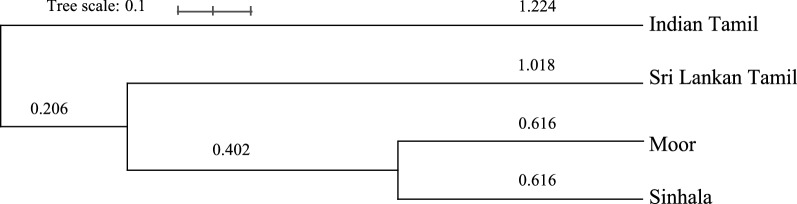
UPGMA phylogram for the four Sri Lankan ethnicities based on 16 X-STR data. The branch lengths are in the same units as those of the evolutionary distances used to infer the phylogenetic tree.

Since phylogenetic trees constructed from genetic distances can easily deduce the evolutionary relationships and origins of different populations^16,17^, UPGMA method was applied for the four ethnicities to further evaluate their genetic affinities. As shown in the phylogram (Fig. 2), Sinhalese and Moors are genetically closely associated with each other and also with Sri Lankan Tamils to a lesser extent. Although Indian Tamil group is placed at a distant position from Moors and Sinhalese, the close genetic affinity between the two Tamil groups are apparent in the phylogram. In addition, the phylogram also supports an Indian origin for the Sri Lankan Moors as suggested by some historians^5^. Nei genetic distances for the six pairwise ethnic groups are listed in the Table 3.

**Table 3:** Nei genetic distances among different pairwise ethnic groups.

Pairwise population	Nei genetic distances
Sinhala-SL Tamil	0.0174
Sinhala-IN Tamil	0.0205
Sinhala-Moors	0.0123
SL Tamil-IN Tamil	0.0237
SL Tamil-Moors	0.0233
IN Tamil-Moors	0.0293

These findings agree with the historical data on early settlement of the four ethnic groups in Sri Lanka. According to anthropological and archaeological evidence, Sri Lankan Tamils have a very long history in Sri Lanka and have lived in the island since at least around the second century BCE. They have arrived in Sri Lanka from various parts of the Indian subcontinent, either with their original families or alone, and subsequently uniting with the Sinhalese through matrimonial bonds. Indian Tamils on the other hand were brought to Sri Lanka to work in estates during the British colonization and had minimum admixture with the native Sinhalese or with Sri Lankan Tamils, who were more economically independent at the time and had better social status. Even at present, majority of Indian Tamils are congregated around the plantation estates of central hill area of the country forming a separate community. In contrast, Sri Lankan Moors have descended exclusively from Muslim male merchants of either Arabic or of Indian origin^5^, who came to Sri Lanka for trading. During the fourteenth century, they started to settle in coastal areas in Sri Lanka and espoused local women, who were either Sinhalese or Sri Lankan Tamil. Thus, it is not surprising to see this local female ancestry reflected among Moors via our X-STR analysis, despite their Arabic origin, in the light that the X chromosome spends two third of its lifetime within females. Alternatively, the genetic similarity observed between Sri Lankan Tamils and Moors may be reflecting the Indian origin of Moors as some scholars claims it to be^5^. Likewise, the genetic similarity observed between the two Tamil populations might also lie in their common Indian origin.

Since the X chromosome reflect more of the maternal blood line, results reported in mitochondrial (mt) DNA studies are also of much relevance to the present context. Despite that the number of mt DNA studies conducted on Sri Lankan ethnicities are very limited, the available reports closely agree with our observations and indicated a very fine genetic structure with a more closer genetic relationship among Sinhalese and Sri Lankan Tamils, in comparison to that among Sinhalese and Indian Tamils^8^. In addition, haplotype sharing between Sinhalese and Moors has also been demonstrated^9^. When taken together with the fact that autosomal STR analysis have failed in detecting a genetic structure among the four ethnic groups^6^, our results suggest a sex-biased demographic history for Sri Lankan ethnicities.

### Genetic distance between Sri Lankan ethnicities and other world populations

In order to understand the genetic composition of Sri Lankan ethnicities in relation to other global populations, allele frequencies generated in the present study were compared with 17 other world populations. Depending on the availability of data in the literature, 8–16 common X-STR markers were used for the analysis. Accordingly, three populations from South Asia (Bhil tribe and Brahmin caste in India^18,19^, Bangladesh^20^ and Pakistan^21,22^), two from South East Asia (Malaysians^23^and Thailand^24^), three from East Asia (China^25–27^, Japan^25,28,29^ and Taiwan^30,31^), five from Europe (Germany^32–35^, Italy^36–40^, Sweden^41^, Denmark^42^ and North Portugal^43^) two from Africa (Somalia^42^, and Ivory Coast^44^) and Brazil^45^ were compared (Supplementary Table S14).

Among the three South Asian populations, Bhil tribe and Brahmin caste populations of India are the most geographically proximal populations to Sri Lankan ethnics groups. Both these Indian populations did not show a significant genetic differentiation with the Sri Lankan ethnicities’ with respect to any of the tested loci. Similarly, Bangladesh population also did not exhibit any population subdivision with Sri Lankan ethnicities. However, Pakistan population demonstrated significant differentiation from one or more ethnic groups of Sri Lanka at two loci (DXS6789, DXS7424) out of the nine common loci compared.

These results suggest that allele distribution of the four ethnic groups of Sri Lanka is very similar to the two tested Indian populations and the Bangladesh population. Pakistani population also exhibit substantial similarity to Sri Lankan ethnic groups, though not to the same extent of Indian and Bangladesh populations. On the contrary, allelic distribution of many X-STR loci in Sri Lankan ethnic groups differ from Southeast Asian, East Asian, European and African populations. Among them, East Asian and African populations are the most genetically distant populations to Sri Lankans.

**Figure 3:**
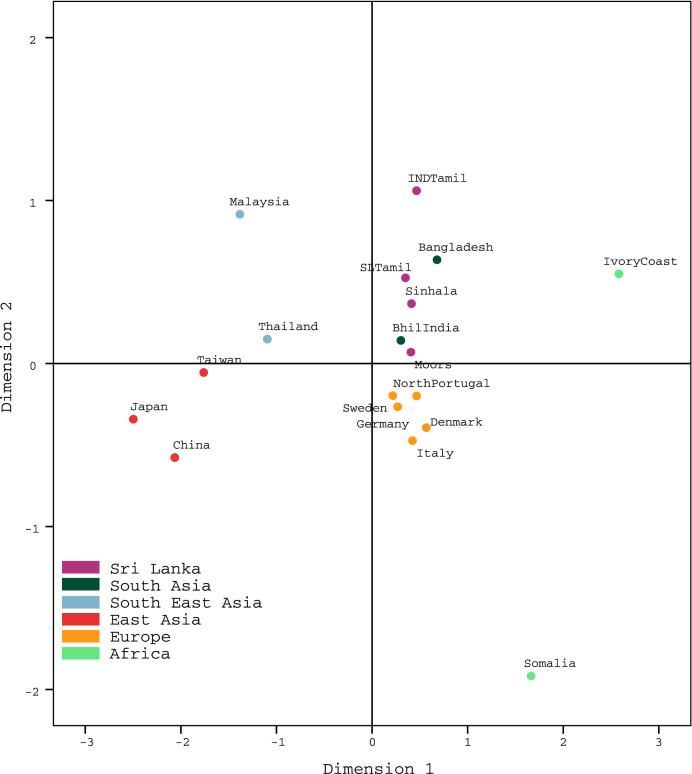
Two-dimensional MDS plot drawn from pairwise Fst values averaged over eight X-STR loci.

To further clarify the relationship between Sri Lankans and the world populations, pairwise Fst values were averaged over eight of the 16 studied X-STR loci (DXS10148, DXS10135, DXS8378, DXS7132, DXS10079, DXS10074, HPRTB and DXS7423) and were represented in a multidimensional scaling (MDS) plot (Fig. 3). The plot graphically illustrates the level of similarity between 18 world populations including the four Sri Lanka ethnicities (Sinhalese, SL Tamil, Indian Tamil, Moors, Bhil India^18^, Bangladesh^20^, Malaysia^23^, Thailand^24^, China^25^, Japan^25^, Taiwan^30^, Germany^32^, Italy^36^, Sweden^41^, Denmark^42^, North Portugal^43^, Somalia^42^, and Ivory Coast^44^). According to the results observed, Sri Lankans were clustered together not only with Indians and Bangladeshi, but also with Europeans. Indian Tamils were placed towards the periphery of this main cluster, while Southeast Asians, East Asians and Africans were placed at a distant, outside the main cluster.

This presentation of MDS plot aligns well with the historical claims of population movements in Eurasia. Sinhalese are believed to be descended from Indo-Aryans, who set forth from boarders of Caspian and Black sea towards Europe and South Asia, early in the third millennium BC. Accordingly, many scholars hold the view that along with the Indo-Aryan language family, many Europeans and South Asian civilization of today share common genetic background reflecting their Bronze age common ancestors. Tamils on the other hand are believed to have descended from the indigenous people of Indian subcontinent. However, Sri Lankan Tamils have admixed with Sinhalese nearly over two millennia, unlike the Indian Tamils, which might explain their relative positions in the MDS plot.

### LD and haplotype analysis

Population studies of various countries have illustrated that the LD is population specific^42,46,47^ and does not necessarily present between markers with close physical proximity^48,49^. In the present study, Sri Lankan Tamils did not exhibit LD within any of the four clusters, while Sinhalese displayed LD within three of the four studied clusters after adjusting for multiple comparisons (Table 4). Further, in cluster I and IV, LD was detected only among Sinhalese population, and only between a single pair of loci (DXS10135 and DXS8378 in cluster I and DXS7424 and DXS101 in cluster II). The cluster II, which had the highest presentation of LD among the four clusters, displayed a highly significant (*P* = 0.0000) LD between the two marker pairs, DXS10074-XS10075 (in Sinhalese, Indian Tamils and Moors) and DXS7132-DXS10075 (among the Sinhalese). However, there was no LD detected between DXS10079 and DXS10074 among Sinhalese in contrast to the results obtained for our initial study, which was conducted with 120 Sinhalese males^15^. This disparity of observations might have caused by the limitations in the initial study with respect to the sample size requirements specified for LD analysis^50^. In cluster III, none of the markers showed a significant LD (corrected *P* = 0.0167), although there was a marginal LD (*P* = 0.0174) detected between DXS6801 and DXS6809 for Sinhalese population. Further, in addition to the pairwise LD analysis within separate clusters, pairwise LD was also analyzed between markers belonging to cluster III and IV, due to their relative close physical proximity (6.78 cM) compared to the other clusters. However, LD was not detected between any of the marker pairs (*P* > 0.05 for eight out of nine marker pairs) as shown in Table 4.

**Table 4:** *P* values for pairwise linkage disequilibrium results for the four ethnicities.

Loci pair	Sinhala	Sri Lankan Tamil	Indian Tamil	Moors
**Cluster 1**				
DXS10148, DXS10135	0.6029	0.0707	0.1486	0.4051
DXS10148, DXS8378	0.1437	0.8618	0.2928	0.1165
DXS10135, DXS8378	**0.0046**	0.6634	0.2217	0.2390
**Cluster II**				
DXS7132, DXS10079	0.5373	0.0378	0.5372	0.6837
DXS7132, DXS10074	0.0410	0.2924	0.1040	0.2472
DXS10079, DXS10074	0.1029	0.0661	0.0202	0.7661
DXS7132, DXS10075	**0.0000**	0.3472	0.2258	0.2234
DXS10079, DXS10075	0.7620	0.1582	0.0197	0.2328
DXS10074, DXS10075	**0.0000**	0.3612	**0.0000**	**0.0000**
**Cluster III**				
DXS6801, DXS6809	0.0174	0.8943	0.4792	0.0251
DXS6801, DXS6789	0.1241	0.6836	0.9615	0.0285
DXS6809, DXS6789	0.3075	0.5340	0.7291	0.9068
**Cluster IV**				
DXS7424, DXS101	**0.0159**	0.5544	0.4060	0.2435
DXS7424, DXS7133	0.2616	0.7064	0.5845	0.1790
DXS101, DXS7133	0.0680	0.0972	0.9476	0.5594
**Cluster III and IV**				
DXS6801, DXS7424	0.5476	0.7227	0.0815	0.0198
DXS6809, DXS7424	0.7421	0.2305	0.5808	0.1146
DXS6789, DXS7424	0.6440	0.8464	0.6726	0.1738
DXS6801, DXS101	0.9240	0.4147	0.8663	0.1879
DXS6809, DXS101	0.2092	0.9795	0.7262	0.0803
DXS6789, DXS101	0.3769	0.7291	0.3066	0.3447
DXS6801, DXS7133	0.8799	0.7682	0.3120	0.9418
DXS6809, DXS7133	0.1199	0.5175	0.7853	0.5001
DXS6789, DXS7133	0.2477	0.5693	0.2664	0.3527

Significant *P* values (after correcting for multiple comparisons) are indicated in bold font.

Since the haplotypes defined by those loci which are found to be in LD tend to behave as alleles, forensic efficiency parameters were calculated based on their observed haplotype frequencies and are given in Table 5. As shown PIC, PD_m_ and PD_f_ values for all these haplotypes were greater than 0.9 while MEC values ranged above 0.8.

**Table 5:** Forensic statistical parameters of the four haplotypes.

Haplotype	Ethnic group	PIC	He	HD	PD_f_	PD_m_	MEC_Kru_	MEC_Des-trio_	MEC_Des-duo_
DXS10135–DXS8378	Sinhala	0.9730	0.9736	0.9774	0.9987	0.9736	0.9478	0.9730	0.9484
DXS7132–DXS10074–DXS10075	Sinhala	0.9753	0.9758	0.9796	0.9989	0.9758	0.9536	0.9753	0.9526
DXS7424–DXS101	Sinhala	0.9608	0.9621	0.9659	0.9973	0.9621	0.9250	0.9608	0.9264
DXS10074–DXS10075	INT	0.9147	0.9201	0.9350	0.9882	0.9201	0.8393	0.9147	0.8484
	Moors	0.9247	0.9289	0.9430	0.9907	0.9289	0.8576	0.9247	0.8649

*PIC* polymorphism information content, *He* expected heterozygosity, *HD* haplotype diversity, *PD*_*f*_ power of discrimination in females, *PD*_*m*_ power of discrimination in males, *MEC*_*Kru*_ mean exclusion chance Kruger, *MEC*_*Des-trio*_ mean exclusion chance Desmaris trio, *MEC*_*Des-duo*_ mean exclusion chance Desmaris duo, *INT* Indian Tamil.

LD is generally expected to be high in populations, which are either small, reproductively isolated or having low population growth^48^. Indian Tamils who are tea estate workers, restricted mostly to central part of the country fits well into this description. In Sri Lanka, there are about 850,000 Indian Tamils, which had very limited genetic mixing with other populations due to social and cultural reasons. Nevertheless, only one loci pair among Indian Tamils showed LD according to our results. As pointed out by Kling et al.^50^, to acquire adequate power to detect LD in X-STR analysis, sample sizes over 200 are often needed. When considering the limited number of Indian Tamil samples used in the present study in calculating LD (63 males), it is possible that the low sample number to have masked the actual existence in LD among Indian Tamils. The same limitation is also valid for Sri Lankan Tamil and Moor ethnicities in which similarly low level of LD were detected. On the other hand, analysing a small sample from a large population can create LD, even among loci which are in linkage equilibrium, due to the under-representation of haplotypes^51^. In this light, it is advisable to increase the number of Sinhalese samples used for LD calculations (258 males in the present study), considering that there are about 15 million Sinhalese in the country. On the other hand, the high level of LD observed within Sinhalese might have resulted from its largely admixed nature. All these facts highlight the requirement of a more exhaustive data set with increased power to detect LD, before concluding on the true pattern of LD among Sri Lankan ethnicities.

In general, the LD reported earlier for South Asian and South East Asian populations like Bangladeshi^20^, Indian Bhil tribe^18^ and Malaysians^23^ was quite low; i.e. no LD was reported among cluster I (DXS10148, DXS1035 and DXS8378) or in cluster II markers (DXS7132, DXS10079 and DXS10074). The marker, DXS10075, in which LD was detected with DXS7132 for Sinhalese was not investigated in any of these populations to make a comparison. However, it is noteworthy that all these studies were conducted with male samples between 100 and160, which might have posed a limitation in detecting true LD in these populations. On the other hand, LD was not observed for cluster III or cluster IV in a study that investigated 302 Pakistan males^21^, the only Asian population for which LD data are available for these clusters. These data are suggestive of the existence of a complex pattern of LD among Asians. In contrast, a high level of LD was reported within these four clusters for Europeans like Swedish^52^ and German^53–55^ populations, in studies conducted with 450–800 male individuals.

The genetic stability of the linked clusters and the degree of dependence between them can have a major influence on most forensic and kinship applications. The classical approach of studying such linkage between selected markers is via analysis of three generation pedigrees using LOD scores^56^. However, a linkage analysis was not conducted for the 16 X-STR markers investigated in the current study. Nevertheless, a previous research conducted for three generation families of Chinese origin has reported a significant linkage with maximum LOD scores > 2.0 for all pairwise markers in the cluster II, III and IV indicating their tightly linked nature^57^.

The haplotype frequencies obtained for the four ethnic groups for all four clusters are listed in Supplementary Tables S15–S18. The forensic efficiency parameters of these haplotypes are also provided in Table S19. In most of the previously published population studies, cluster 1 V comprises only DXS7424-DXS101 without DXS7133^55,58,59^. Likewise, the four markers included in the cluster II had been analyzed in two different combinations; i.e. DXS7132-DXS10079-DXS10074 (Argus X-12 kit) and DXS10079-DXS10074-DXS10075^54^. Therefore, to allow comparisons with these previous studies, the haplotype frequencies of above combinations are also listed in Supplementary Tables S20–S23.

As shown in Supplementary Table S24, typing of male subsamples from the four ethnic groups yielded a total of 604, 246, 208 and 223 different haplotypes for Sinhalese, Sri Lankan Tamils, Indian Tamils and Moors, respectively. Cluster I produced the highest number of haplotypes for Sinhalese and Sri Lankan Tamils, while both cluster I and II produced the highest number of haplotypes for Indian Tamils and Moors. Among all the observed haplotypes, 96.85% of Sinhalese, 82.11% of Sri Lankan Tamil, 83.17% of Indian Tamil and 85.65% of Moor haplotypes showed frequencies < 0.020. Moreover, the most common haplotype was observed at a frequency ≤ 0.065 in all the four ethnicities, indicating the suitability of the selected X-STR clusters for haplotype based kinship analysis.

Among the four clusters, both clusters I and II proved relatively more informative for all four ethnicities as reflected by the haplotype diversity (HD = 0.9964–0.9987 and 0.9935–0.9973 respectively). In cluster I, this observation may have caused by the two highly polymorphic markers, DXS10135 and DXS10148 as described above and is consistent with other previously published population data^18,20,25^. Cluster II, on the other hand carries four markers compared to the other clusters, which consists of three markers each. This increased number of markers may have generated higher haplotype diversity with respect to cluster II among the studied populations. On the contrary, cluster III and cluster IV are equally informative (HD = 0.9873–0.9908 and 0.9882–0.9923 respectively), though not to the same extent as the clusters I and II. Nevertheless, in general, all four clusters showed a high haplotype diversity for all four ethnicities.

### Combined forensic efficiency parameter data

In general, for those loci in LD, the combined forensic parameters are calculated based on the haplotypes defined by LD. However, a more conservative approach would be better suited in the current scenario, considering the limitations posed by the number of samples in calculating LD. Accordingly, the combined forensic efficiency parameters for the 16 X-STR were calculated based on the efficiency of haplotypes defined by the four clusters. In addition, the efficiency of the three individual markers were taken separately. The combined power of discrimination for both males (CPD_m_ > 0.999999991334440) and females (CPD_f_ > 0.999999999999996) as well as combined MEC indices calculated for deficiency (CMEC_Kru_ > 0.999999242880176), normal trio (CMEC_Des-trio_ > 0.999999984443189) and duo cases (CMEC_Des-duo_ > 0.999999435330137) were equally high for all four ethnicities (Table 6). These results suggest that the 16 tested X-STR loci are appropriate candidates for kinship and forensic analysis among the four Sri Lankan ethnicities, especially with the cases involving female offspring.

**Table 6:** Combined forensic efficiency parameters calculated for the 16 X-STR loci in the four ethnic groups based on the haplotypes frequencies of the four clusters and the allele frequencies of the three individual markers.

	Sinhalese	SLT	INT	Moors
CPD_f_	1.000 000 000 000 000	0.999 999 999 999 999	0.999 999 999 999 996	0.999 999 999 999 997
CPD_m_	0.999 999 999 744 418	0.999 999 999 578 340	0.999 999 991 334 440	0.999 999 992 982 243
CMEC_Kru_	0.999 999 988 803 328	0.999 999 586 011 089	0.999 999 242 880 176	0.999 999 278 503 647
CMEC_Des-trio_	0.999 999 999 476 738	0.999 999 991 970 915	0.999 999 984 443 189	0.999 999 987 537 770
CMEC_Des-duo_	0.999 999 978 007 651	0.999 999 691 336 852	0.999 999 435 330 137	0.999 999 545 624 459

*CPD*_*f*_ combined power of discrimination in females, *CPD*_*m*_ combined power of discrimination in males, *CMECKru* combined mean exclusion chance Kruger, *CMEC*_*Des-trio*_ combined mean exclusion chance Desmaris trio, *CMEC*_*Des-duo*_ combined mean exclusion chance Desmaris duo, *SLT* Sri Lankan Tamil, *INT* Indian Tamil.

## Conclusion

In this work, we report for the first time, X chromosome based population genetic data for all four major ethnic groups in Sri Lanka which covers 99.5% of the total population. According to our results, the 16 X-STR assay system used in the current study is highly polymorphic and exhibited high forensic efficiency for all four ethnicities tested indicating its suitability to be used in both evolutionary genetic analysis and forensic applications of Sri Lankans. The present study has also revealed subtle but statistically significant differences in X-STR based allele frequency distribution of Indian Tamils with Sinhalese and Moors, contrary to the highly homogeneous genetic outlook portrayed by autosomal STR analysis. While suggesting a sex biased demographic history for Sri Lankan ethnicities, this observation recommends the use of a separate X-STR allele frequency database for Indian Tamils for forensic and kinship application purposes. In contrast, the observed genetic admixture present within Sinhalese, Sri Lankan Tamil and Moor ethnicities suggest the possible use of a common allele database for the purpose. Further, the genetic distances observed among the Sri Lankans and other nationalities in the world visualized in the MDS plot render evidence to the ancient linguistic origin of Sri Lankan ethnicities—Indo-Aryan origin of Sinhalese and Dravidian origin of Tamil populations—which had been later affected to various degrees through genetic admixing between them. LD was detected along the X chromosome in all ethnic groups except Sri Lankan Tamils, which need to be considered during the likelihood calculations of kinship resolution and person identification. Further, the patterns of LD observed, which differ substantially among the four ethnicities request the use of different haplotype frequency databases for the four ethnicities for forensic purposes. Although the results of haplotype analysis suggest that the four studied X-STR clusters can provide a powerful tool for kinship testing and relationship identification of Sri Lankan ethnicities, a more exhaustive sampling of the two Tamil groups and Moors is recommended to confirm the LD and haplotype based observations.

## Methods

### Sample preparation and DNA extraction

The study was conducted with the approval of the Ethics Review Committee, Institute of Biology, Sri Lanka (ERC IOBSL 135 11 15) and the study was performed in line with the principles of the Declaration of Helsinki. Written informed consent was obtained from all individual participants included in the study. Finger pricked blood samples were collected from 838 unrelated individuals from the four ethnic groups in the Sri Lankan population; 426 samples from Sinhalese (60.6% males), 154 samples from the Sri Lankan Tamils (50% males), 128 samples from Indian Tamils (49.2% males), and 130 samples from Sri Lankan Moors (51.5% males). Genomic DNA was extracted using Chelex-100 method^60^ and subjected to PCR amplification using the single tube 16 X-STR multiplex system described in Perera et al.^15^. Amplified products were resolved with capillary gel electrophoresis using ABI 3500 Genetic Analyzer (Thermo Fisher Scientific, USA) and data analysis allele designation was performed using GeneMapper IDX software (Thermo Fisher Scientific, USA).

### Statistical analysis

All population genetic parameters were calculated using ARLEQUIN 3.5. Allele and haplotype frequencies, exact test of differentiation for male and female allele frequencies, conformity of female subsamples to Hardy–Weinberg equilibrium and pairwise test of LD between pairs of markers within clusters of linked loci in the male sub samples were analysed separately for all four ethnic groups. LD between the markers of cluster III and IV was also analysed, considering the close physical proximity of the two clusters (6.78 cM). Forensic parameters, i.e. polymorphism information content (PIC), expected heterozygosity (He), mean exclusion chance in deficiency (MEC_Kruger_), mean exclusion chance in Duos (MEC_Desmaris duo_), mean exclusion chance in trios (MEC_Desmaris trio_), power of discrimination for females (PD_f_), power of discrimination for males (PD_m_) were calculated using chromosome X web (http://www.chrx-str.org) for all X-STR loci and haplotypes defined by the four clusters using MATLAB software (version R2017a) for all four ethnic groups. For those loci under LD, the parameters were also evaluated based on frequencies of haplotypes defined by them using the latter software.

Locus-by-locus analysis of molecular variance (AMOVA) was conducted by grouping the four ethnicities based on their linguistic origin; i.e. Indo-Aryan origin (Sinhalese) versus Dravidian origin (Tamils and Moors) to create a hierarchical structure. Nei’s average number of pairwise differences within and between populations was used to detect pairwise differences among the four ethnic groups. The null distribution of pairwise Fst values was obtained by permuting haplotypes between populations for 1000 times. The significance level was kept at 0.05 and all analyses were conducted using Arlequin software v.3.5.1.2^61^. Further, for easier visualization of the observed genetic distances, a phylogenetic tree was also constructed with Molecular Evolutionary Genetics Analysis Version 6.0 (MEGA 6.0)^62^ software using the Unweighted Pair Group Method with Arithmetic mean (UPGMA) method. The reliability of phylograms was estimated by bootstrapping 2000 replicates over loci and the extended majority rule consensus trees were inferred. To compare the Sri Lankan ethnicities with populations from other countries in the world, locus by locus pairwise genetic distances (Fst) were generated based on data available in the literature. Resulting Fst values were averaged over loci and represented in a multidimensional scaling (MDS) plot using IBM SPSS Statistics Version 21 statistical package^63^.

## Supplementary Information

Supplementary Information.

## Data Availability

The datasets generated during the current study are available from the corresponding author on reasonable request.
