# Author Correction: X-chromosomal STR based genetic polymorphisms and demographic history of Sri Lankan ethnicities and their relationship with global populations

**DOI:** 10.1038/s41598-021-97492-0

**Published:** 2021-09-01

**Authors:** Nandika Perera, Gayani Galhena, Gaya Ranawaka

**Affiliations:** 1 Genetech Molecular Diagnostics, Colombo 08, Sri Lanka; 2 Faculty of Health Sciences, The Open University of Sri Lanka, Nawala, Sri Lanka; 3 Department of Zoology and Environment Sciences, University of Colombo, Colombo 03, Sri Lanka

Correction to: *Scientific Reports* 10.1038/s41598-021-92314-9, published online 17 June 2021

The original version of this Article contained errors in Figure 2, where the labels showing the four Sri Lankan ethnicities were omitted.

The original Figure 2 and accompanying legend appear below.

**Figure 2:**
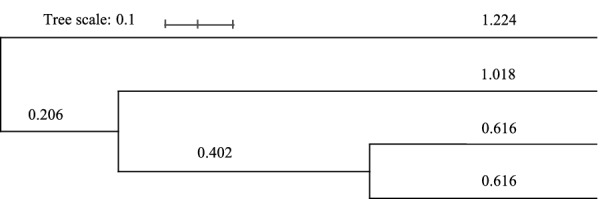
UPGMA phylogram for the four Sri Lankan ethnicities based on 16 X-STR data. The branch lengths are in the same units as those of the evolutionary distances used to infer the phylogenetic tree.

The original Article has been corrected.

