# Author Correction: X-chromosomal STR based genetic polymorphisms and demographic history of Sri Lankan ethnicities and their relationship with global populations

**DOI:** 10.1038/s41598-021-00040-z

**Published:** 2021-10-12

**Authors:** Nandika Perera, Gayani Galhena, Gaya Ranawaka

**Affiliations:** 1 Genetech Molecular Diagnostics, Colombo 08, Sri Lanka; 2 Faculty of Health Sciences, The Open University of Sri Lanka, Nawala, Sri Lanka; 3 Department of Zoology and Environment Sciences, University of Colombo, Colombo 03, Sri Lanka

Correction to: *Scientific Reports* https://doi.org/10.1038/s41598-021-92314-9, published online 17 June 2021

The original version of this Article contained an error, as the paper has erroneously stated that there were no mtDNA studies conducted on Moors previously. As a result, Reference 9 was omitted and is listed below,

Ranasinghe, R., Tennekoon, K. H., Karunanayake, E. H., Lembring, M. & Allen, M. A study of genetic polymorphisms in mitochondrial DNA hypervariable regions I and II of the five major ethnic groups and Vedda population in Sri Lanka. *Leg*. *Med*. *(Tokyo)* **17**, 539–546, https://doi.org/10.1016/j.legalmed.2015.05.007 (2015).

Consequently, in the Introduction,

“Although a few previous studies that had been conducted to understand the genetic substructure and underlying heterogeneity of Sri Lankan ethnicities using autosomal^6^, Y chromosomal^7^ and mitochondrial markers^8^, none has utilized those present on the X chromosome.”

now reads:

“Although a few previous studies that had been conducted to understand the genetic substructure and underlying heterogeneity of Sri Lankan ethnicities using autosomal^6^, Y chromosomal^7^ and mitochondrial markers^8,9^, none has utilized those present on the X chromosome.”

In the Results and discussion section, under the subheading “Population differentiation among Sri Lankan ethnicities”,

“Although there are no mtDNA studies conducted on Moors, research on the other three ethnic groups have reported the existence of a very fine genetic structure with a more closer genetic relationship among Sinhalese and Sri Lankan Tamils, in comparison to that among Sinhalese and Indian Tamils^8^.”

now reads:

“Despite that the number of mt DNA studies conducted on Sri Lankan ethnicities are very limited, the available reports closely agree with our observations and indicated a very fine genetic structure with a more closer genetic relationship among Sinhalese and Sri Lankan Tamils, in comparison to that among Sinhalese and Indian Tamils^8^. In addition, haplotype sharing between Sinhalese and Moors has also been demonstrated^9^.”

All the subsequent references have been renumbered.

The original Article has been corrected.

