# Supplementary material for: X-chromosomal STR based genetic polymorphisms and demographic history of Sri Lankan ethnicities and their relationship with global populations
Source: Sci Rep. 2021 Jun 17;11:12748. doi: 10.1038/s41598-021-92314-9 (PMC8211843; doi:10.1038/s41598-021-92314-9)
Supplement: Supplementary file 1 — Supplementary Information. [file 41598_2021_92314_MOESM1_ESM.pdf]

Supplementary Figure S1 and Supplementary Tables S1 – S24

X-chromosomal STR based genetic polymorphisms and demographic history of Sri Lankan ethnicities and their relationship with global populations

Nandika Perera[1,2], Gayani Galhena[3*], Gaya Ranawaka[2]

[1]Genetech Molecular Diagnostics, Colombo 08, Sri Lanka
[2]Faculty of Health Sciences, The Open University of Sri Lanka, Nawala, Sri Lanka
[3]Department of Zoology and Environment Sciences, University of Colombo, Colombo 03, Sri Lanka

*Correspondence and requests for materials should be addressed to gayanihg@gmail.com

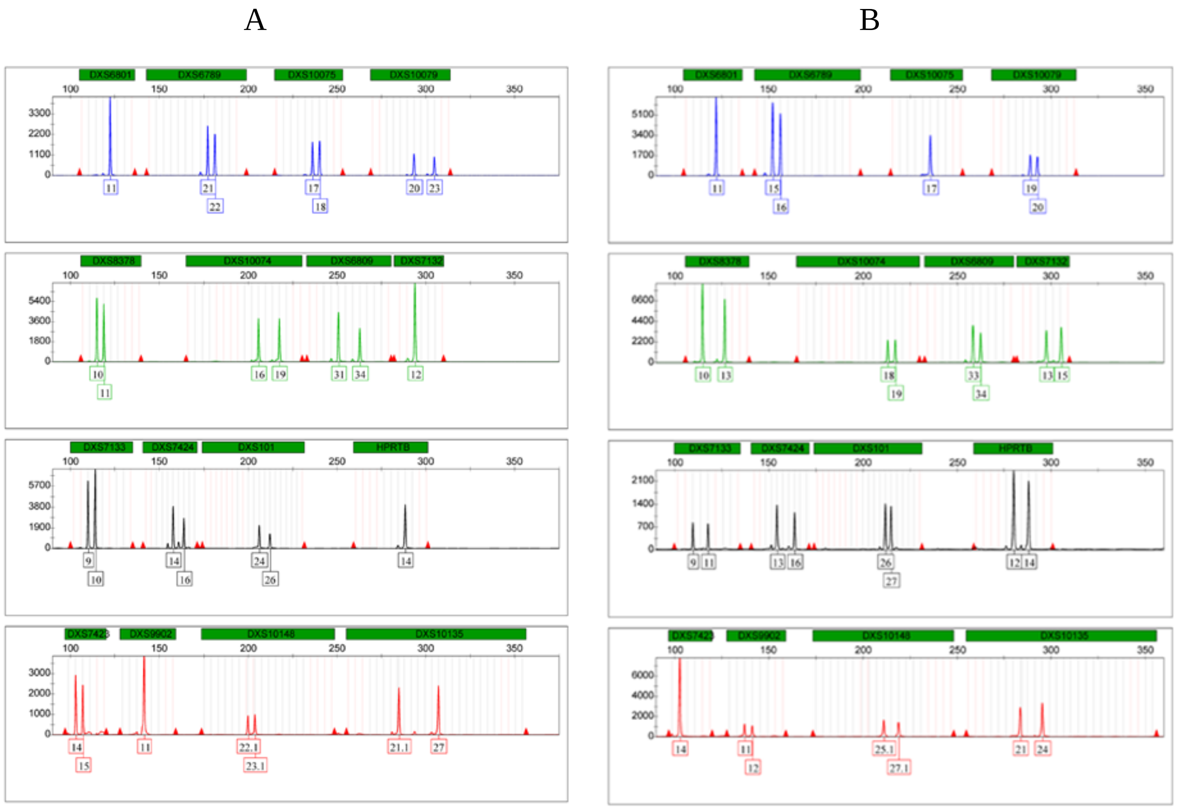

Supplementary Figure S1. Electropherograms generated for 9947A control DNA (A) and for a female sample (B) for the 16 X-STR multiplex system

Supplementary Table S1. Observed and expected heterozygosities and Hardy Weinberg P values for the 16 X-STR loci for female samples of the four ethnicities

	Sinhala			SL Tamil			IND Tamil			Moors		
Locus	Obs. Het.	Exp. Het.	P-value	Obs. Het.	Exp. Het.	P-value	Obs. Het.	Exp. Het.	P-value	Obs. Het.	Exp. Het.	P-value
DXS10148	0.8393	0.8966	0.2658	0.8831	0.9014	0.1870	0.7539	0.8893	0.0086	0.8889	0.9016	0.6999
DXS10135	0.9286	0.9340	0.2306	0.8831	0.9363	0.1195	0.9231	0.9238	0.7747	0.9365	0.9308	0.3653
DXS8378	0.6548	0.7007	0.0545	0.6234	0.6825	0.4936	0.6000	0.6297	0.4633	0.7143	0.6994	0.5698
DXS9902	0.6607	0.7411	0.2868	0.7403	0.7560	0.8015	0.6769	0.7232	0.7970	0.6191	0.7366	0.1727
DXS7132	0.7738	0.7347	0.8160	0.6883	0.7419	0.3672	0.7539	0.7419	0.4683	0.7143	0.7432	0.6338
DXS10079	0.8333	0.8267	0.0121	0.7792	0.8180	0.4277	0.8000	0.8222	0.2245	0.8413	0.8319	0.8996
DXS10074	0.8095	0.7965	0.5550	0.7662	0.8257	0.2216	0.9231	0.8032	0.6866	0.8095	0.8249	0.5943
DXS10075	0.6488	0.6732	0.2660	0.7662	0.6997	0.6506	0.7231	0.7350	0.9893	0.7460	0.7209	0.9704
DXS6801	0.6131	0.6684	0.2069	0.5844	0.6397	0.2035	0.7231	0.7026	0.0785	0.6667	0.6702	0.7545
DXS6809	0.7262	0.8062	0.0194	0.7533	0.8131	0.0839	0.8462	0.8171	0.1398	0.8571	0.7977	0.8134
DXS6789	0.8274	0.7829	0.7141	0.7922	0.7841	0.5919	0.6923	0.7946	0.0548	0.7778	0.8151	0.9414
DXS7424	0.8274	0.8369	0.0123	0.7792	0.8202	0.6334	0.7692	0.7764	0.3223	0.8095	0.8258	0.4403
DXS101	0.8095	0.8188	0.1392	0.7403	0.8498	0.0093	0.8462	0.8464	0.2796	0.8413	0.8156	0.5327
DXS7133	0.6964	0.6550	0.1544	0.5325	0.5826	0.3662	0.5385	0.5541	0.6216	0.6032	0.6340	0.0328
HPRTB	0.7262	0.7429	0.9043	0.6234	0.7304	0.1846	0.7539	0.7137	0.4004	0.6825	0.7459	0.1289
DXS7423	0.6845	0.6541	0.7186	0.6234	0.5949	0.7301	0.6308	0.5819	0.8060	0.5556	0.6318	0.1338

Supplementary Table S2. Allele frequencies and forensic parameters of DXS10148, DXS10135, DXS8378 and DXS9902 for four ethnicities

Allele	DXS10148				DXS10135				DXS8378				DXS9902			
	Sinhala	SL Tamil	IND Tamil	Moors	Sinhala	SL Tamil	IND Tamil	Moors	Sinhala	SL Tamil	IND Tamil	Moors	Sinhala	SL Tamil	IND Tamil	Moors
6													0.0017	0.0043		0.0052
7																
8									0.0051					0.0043	0.0104	
9									0.0690	0.0303	0.0570	0.0415	0.1263	0.1602	0.0881	0.0881
10									0.2340	0.2381	0.1865	0.2902	0.3233	0.3030	0.2694	0.3109
11									0.4276	0.4416	0.5233	0.4249	0.3148	0.3074	0.4197	0.2798
11.1													0.0017		0.0104	
12									0.2340	0.2511	0.1917	0.2124	0.2037	0.1905	0.1814	0.2902
12.1													0.0051	0.0087		0.0052
13									0.0286	0.0260	0.0415	0.0311	0.0219	0.0217	0.0207	0.0207
14		0.0043			0.0017				0.0017	0.0130			0.0017			
15																
16			0.0052		0.0017	0.0087										
17	0.0269	0.0260	0.0207	0.0052	0.0168	0.0217		0.0104								
18	0.1599	0.1515	0.2021	0.1554	0.0269	0.0390	0.0259	0.0363								
19	0.0455	0.0520	0.0518	0.0622	0.0337	0.0606	0.0363	0.0311								
19.1				0.0155												
20	0.0505	0.0433	0.0829	0.0725	0.0640	0.0476	0.0415	0.0518								
20.1	0.0101	0.0130		0.0104				0.0052								
21	0.0471	0.0390	0.0518	0.0311	0.0724	0.0346	0.0570	0.0674								
21.1	0.0101	0.0130	0.0104	0.0155	0.0017	0.0087		0.0155								
22	0.0067	0.0087	0.0104		0.0960	0.0996	0.0622	0.1036								
22.1	0.0202	0.0087	0.0155	0.0311		0.0043										
23		0.0087	0.0052	0.0052	0.0724	0.0693	0.0415	0.0725								
23.1	0.0337	0.0520	0.0518	0.0363												
23.2	0.0017															
24				0.0052	0.0673	0.0693	0.0881	0.0363								
24.1	0.1364	0.0649	0.0622	0.0829												
25	0.0017				0.0791	0.0693	0.0674	0.0881								
25.1	0.1195	0.1602	0.1192	0.1658												
26	0.0017	0.0043			0.0909	0.0703	0.1244	0.0466								

Allele	DXS10148				DXS10135				DXS8378				DXS9902			
	Sinhala	SL Tamil	IND Tamil	Moors	Sinhala	SL Tamil	IND Tamil	Moors	Sinhala	SL Tamil	IND Tamil	Moors	Sinhala	SL Tamil	IND Tamil	Moors
26.1	0.1465	0.1775	0.1244	0.1814												
26.2	0.0017															
27					0.0976	0.0909	0.1088	0.1088								
27.1	0.0909	0.1039	0.1244	0.0570												
28					0.0774	0.0606	0.0622	0.0674								
28.1	0.0673	0.0346	0.0363	0.0466												
29					0.0791	0.0649	0.0985	0.1244								
29.1	0.0152	0.0217	0.0259	0.0155												
29.2				0.0052		0.0043										
30					0.0320	0.0433	0.0777	0.0207								
30.1	0.0051	0.0130														
31					0.0337	0.0563	0.0311	0.0674								
31.1	0.0017															
32					0.0152	0.0390	0.0155	0.0104								
33					0.0118	0.0043	0.0259	0.0155								
34					0.0118		0.0155	0.0104								
35					0.0067	0.0087	0.0155	0.0104								
36																
37					0.0067	0.0043										
38					0.0017	0.0043	0.0052									
39					0.0017	0.0087										

Supplementary Table S3. Allele frequencies of DXS7132, DXS10079, DXS10074 and DXS10075 for four ethnicities

Allele	DXS7132				DXS10079				DXS10074				DXS10075			
	Sinhala	SL Tamil	IND Tamil	Moors	Sinhala	SL Tamil	IND Tamil	Moors	Sinhala	SL Tamil	IND Tamil	Moors	Sinhala	SL Tamil	IND Tamil	Moors
5											0.0052					
7									0.0253	0.0390	0.0415	0.0415				
8									0.0236	0.0217	0.0259	0.0466				
9									0.0051	0.0130	0.0104	0.0259				
11	0.0101	0.0087	0.0207							0.0043						
12	0.1162	0.0996	0.0985	0.1503					0.0017	0.0043	0.0052		0.0034		0.0052	0.0104
13	0.2929	0.2597	0.2539	0.2798	0.0017	0.0087							0.0253	0.0390	0.0415	0.0311
13.2																
14	0.3771	0.3853	0.3420	0.3575		0.0043	0.0052		0.0101		0.0207	0.0155	0.0067	0.0217	0.0155	0.0415
15	0.1700	0.2035	0.2487	0.1658	0.0152	0.0087	0.0207	0.0104	0.1027	0.1299	0.0674	0.0777	0.0202	0.0087	0.0207	0.0207
15.3									0.0017							
16	0.0337	0.0390	0.0311	0.0259	0.0438	0.0260	0.0155	0.0311	0.2492	0.2165	0.2176	0.2021	0.2138	0.2251	0.2124	0.1917
16.1										0.0043						
17		0.0043	0.0052	0.0207	0.0606	0.0779	0.0777	0.0829	0.2778	0.2597	0.2643	0.2953	0.4562	0.4762	0.4352	0.4145
17.1									0.0017							
17.3									0.0017						0.0052	
18					0.1431	0.0996	0.1295	0.2021	0.2003	0.2165	0.2435	0.1865	0.2441	0.2165	0.2228	0.2643
19					0.2273	0.2511	0.2435	0.2124	0.0791	0.0779	0.0829	0.0881	0.0286	0.0130	0.0311	0.0259
19.3					0.0067											
20					0.2727	0.2544	0.3005	0.2383	0.0168	0.0130	0.0155	0.0207	0.0017		0.0052	
20.1							0.0052									
21					0.1515	0.1991	0.0052	0.1606	0.0034						0.0052	
21.1					0.0017			0.0052								
22					0.0640	0.0476	0.0570	0.0518								
23					0.0101	0.0217	0.0155	0.0052								
24					0.0017		0.0052									

Supplementary Table S4. Allele frequencies of DXS6801, DXS6809, DXS6789 and DXS7424 for four ethnicities

Allele	DXS6801				DXS6809				DXS6789				DXS7424			
	Sinhala	SL Tamil	IND Tamil	Moors	Sinhala	SL Tamil	IND Tamil	Moors	Sinhala	SL Tamil	IND Tamil	Moors	Sinhala	SL Tamil	IND Tamil	Moors
8	0.0017	0.0130	0.0052	0.0052												
9	0.0051	0.0130	0.0207	0.0052												
10	0.2323	0.2078	0.3161	0.2539									0.0101	0.0043		
11	0.4613	0.4849	0.4042	0.4301									0.0960	0.1082	0.0466	0.0985
12	0.2306	0.1991	0.1969	0.2383									0.0606	0.0606	0.0155	0.0829
13	0.0640	0.0693	0.0570	0.0674									0.2458	0.2597	0.2694	0.2073
14	0.0051	0.0130							0.0303	0.0173	0.0259	0.0155	0.1684	0.2338	0.2539	0.1969
15									0.2206	0.2900	0.2902	0.2591	0.1953	0.2165	0.2694	0.2228
16									0.0859	0.1299	0.0829	0.1036	0.1717	0.1039	0.0933	0.1399
17									0.0202	0.0130	0.0155	0.0207	0.0421	0.0087	0.0363	0.0518
18									0.0101		0.0259	0.0155	0.0084	0.0043	0.0155	
19									0.0067	0.0173	0.0155	0.0415	0.0017			
20									0.3367	0.2944	0.3109	0.3005				
21									0.1684	0.1255	0.1503	0.1451				
22									0.0909	0.0866	0.0363	0.0674				
23									0.0286	0.0260	0.0311	0.0259				
24									0.0017		0.0155	0.0052				
27					0.0017	0.0087										
28					0.0051	0.0130	0.0104									
29					0.0152	0.0217	0.0311	0.0207								
30					0.0337	0.0173	0.0415	0.0207								
31					0.1010	0.0823	0.1295	0.1036								
32					0.1179	0.1039	0.1295	0.1554								
33					0.3418	0.3160	0.3264	0.3212								
34					0.2121	0.2165	0.1606	0.2435								
35					0.0943	0.1429	0.1244	0.0829								
36					0.0505	0.0563	0.0415	0.0363								
37					0.0253	0.0217	0.0052	0.0155								
38					0.0017											

Supplementary Table S5. Allele frequencies of DXS101, DXS7133, HPRTB and DXS7423 for four ethnicities

Allele	DXS101				DXS7133				HPRTB				DXS7423			
	Sinhala	SL Tamil	IND Tamil	Moors	Sinhala	SL Tamil	IND Tamil	Moors	Sinhala	SL Tamil	IND Tamil	Moors	Sinhala	SL Tamil	IND Tamil	Moors
**7**					0.0034											
8					0.0404	0.0476	0.0207	0.0155								
9					0.5084	0.5844	0.5803	0.4767								
10					0.1549	0.1558	0.1710	0.1865	0.0051	0.0043						
11					0.2508	0.1688	0.1710	0.2850	0.0640	0.0520	0.0311	0.0674				
11.2									0.0017							
12					0.0118	0.0130	0.0363	0.0155	0.2677	0.2857	0.2228	0.3420				
13					0.0084	0.0173	0.0155	0.0207	0.3519	0.3680	0.4042	0.3161	0.0269	0.0173	0.0311	0.0570
13.2									0.0017							
14					0.0202	0.0130	0.0052		0.2020	0.2251	0.2487	0.1969	0.4478	0.4502	0.4560	0.4255
15	0.0101	0.0087	0.0052		0.0017				0.1044	0.0563	0.0777	0.0622	0.4057	0.4372	0.4508	0.3990
16										0.0087	0.0155	0.0104	0.1094	0.0866	0.0622	0.1036
17									0.0017			0.0052	0.0101	0.0087		0.0155
18	0.0135	0.0130	0.0207	0.0052												
19	0.0017	0.0043	0.0052	0.0052												
20	0.0084	0.0087	0.0104	0.0052												
21	0.0084	0.0173	0.0052	0.0052												
22	0.0303	0.0390	0.0207	0.0259												
23	0.0724	0.1212	0.1451	0.0985												
24	0.2963	0.2121	0.2591	0.2591												
25	0.2205	0.2208	0.1865	0.2539												
26	0.1616	0.1818	0.1658	0.1192												
27	0.0875	0.0866	0.0881	0.1399												
28	0.0724	0.0693	0.0622	0.0570												
29	0.0101	0.0173	0.0207	0.0207												
30	0.0051		0.0052	0.0052												
31	0.0017															

Supplementary Table S6. Forensic parameters of 16 X-STR loci among the four ethnic populations

	DXS10148				DXS10135			
	Sinhala	SLT	INT	Moors	Sinhala	SLT	INT	Moors
PIC	0.8889	0.8838	0.8831	0.8802	0.9260	0.9345	0.9210	0.9206
He	0.8976	0.8929	0.8923	0.8898	0.9304	0.9380	0.9258	0.9255
PD_f	0.9808	0.9794	0.9792	0.9782	0.9907	0.9926	0.9896	0.9895
PD_m	0.8976	0.8929	0.8923	0.8898	0.9304	0.9380	0.9258	0.9255
MEC_{Kru}	0.7940	0.7865	0.7850	0.7803	0.8586	0.8739	0.8498	0.8491
$MEC_{Des\text{-}trio}$	0.8889	0.8838	0.8831	0.8802	0.9260	0.9345	0.9210	0.9206
$MEC_{Des\text{-}duo}$	0.8082	0.8010	0.7997	0.7956	0.8663	0.8803	0.8583	0.8577

Supplementary Table S7. Forensic parameters of 16 X-STR loci among the four ethnic populations

	DXS8378				DXS9902			
	Sinhala	SLT	INT	Moors	Sinhala	SLT	INT	Moors
PIC	0.6526	0.6285	0.6044	0.6314	0.6927	0.7085	0.6633	0.6837
He	0.7020	0.6835	0.6496	0.6874	0.7384	0.7511	0.7099	0.7326
PD_f	0.8618	0.8449	0.8320	0.8463	0.8859	0.8954	0.8692	0.8796
PD_m	0.7020	0.6835	0.6496	0.6874	0.7384	0.7511	0.7099	0.7326
MEC_{Kru}	0.4555	0.4266	0.4119	0.4287	0.4991	0.5187	0.4699	0.4863
$MEC_{Des\text{-}trio}$	0.6526	0.6285	0.6044	0.6314	0.6927	0.7085	0.6633	0.6837
$MEC_{Des\text{-}duo}$	0.5082	0.4825	0.4563	0.4857	0.5523	0.5702	0.5201	0.5423

Supplementary Table S8. Forensic parameters of 16 X-STR loci among the four ethnic populations

	DXS7132				DXS10079			
	Sinhala	SLT	INT	Moors	Sinhala	SLT	INT	Moors
PIC	0.6831	0.6873	0.7026	0.7002	0.79741	0.7873	0.8040	0.7962
He	0.7283	0.7311	0.7456	0.7427	0.82046	0.8125	0.8233	0.8207
PD_f	0.8810	0.8838	0.8923	0.8913	0.94471	0.9396	0.9495	0.9433
PD_m	0.7283	0.7311	0.7456	0.7427	0.82046	0.8125	0.8233	0.8207
MEC_{Kru}	0.4912	0.4966	0.5134	0.5111	0.64806	0.6329	0.5350	0.6433
$MEC_{Des\text{-}trio}$	0.6831	0.6873	0.7026	0.7002	0.79741	0.7873	0.8040	0.7962
$MEC_{Des\text{-}duo}$	0.5419	0.5465	0.5639	0.5610	0.67993	0.6670	0.6910	0.6777

Supplementary Table S9. Forensic parameters of 16 X-STR loci among the four ethnic populations

	DXS10074				DXS10075			
	Sinhala	SLT	INT	Moors	Sinhala	SLT	INT	Moors
PIC	0.7746	0.7881	0.7830	0.7956	0.6341	0.6230	0.6699	0.6888
He	0.8021	0.8136	0.8088	0.8181	0.6846	0.6735	0.7123	0.7301
PD_f	0.9333	0.9397	0.9376	0.9444	0.8500	0.8429	0.8748	0.8858
PD_m	0.8021	0.8136	0.8088	0.8181	0.6846	0.6735	0.7123	0.7301
MEC_{Kru}	0.6145	0.6325	0.6277	0.6474	0.4372	0.4260	0.4824	0.5040
$MEC_{Des\text{-}trio}$	0.7746	0.7881	0.7830	0.7956	0.6341	0.6230	0.6699	0.6888
$MEC_{Des\text{-}duo}$	0.6509	0.6677	0.6619	0.6780	0.4885	0.4766	0.5278	0.5493

Supplementary Table S10. Forensic parameters of 16 X-STR loci among the four ethnic populations

	**DXS6801**				**DXS6809**			
	Sinhala	SLT	INT	Moors	Sinhala	SLT	INT	Moors
PIC	0.6219	0.6311	0.6390	0.6342	0.7769	0.7876	0.7923	0.7655
He	0.6759	0.6768	0.6943	0.6892	0.8006	0.8107	0.8141	0.7933
PD_f	0.8409	0.8498	0.8512	0.8484	0.9365	0.9410	0.9437	0.9294
PD_m	0.6759	0.6768	0.6943	0.6892	0.8006	0.8107	0.8141	0.7933
MEC_{Kru}	0.4201	0.4380	0.4374	0.4309	0.6234	0.6369	0.6436	0.6038
$MEC_{Des\text{-}trio}$	0.6219	0.6311	0.6390	0.6342	0.7769	0.7876	0.7923	0.7655
$MEC_{Des\text{-}duo}$	0.4750	0.4848	0.4940	0.4882	0.6544	0.6678	0.6735	0.6397

Supplementary Table S11. Forensic parameters of 16 X-STR loci among the four ethnic populations

	**DXS6789**				**DXS7424**			
	Sinhala	SLT	INT	Moors	Sinhala	SLT	INT	Moors
PIC	0.7644	0.7577	0.7562	0.7773	0.8063	0.7766	0.7430	0.8072
He	0.7917	0.7876	0.7853	0.8029	0.8288	0.8047	0.7776	0.8298
PD_f	0.9293	0.9250	0.9248	0.9355	0.9482	0.9337	0.9159	0.9484
PD_m	0.7917	0.7876	0.7853	0.8029	0.8288	0.8047	0.7776	0.8298
MEC_{Kru}	0.6035	0.5921	0.5937	0.6215	0.6587	0.6143	0.5684	0.6588
$MEC_{Des\text{-}trio}$	0.7644	0.7577	0.7562	0.7773	0.8063	0.7766	0.7430	0.8072
$MEC_{Des\text{-}duo}$	0.6385	0.6300	0.6292	0.6549	0.6909	0.6526	0.6118	0.6916

Supplementary Table S12. Forensic parameters of 16 X-STR loci among the four ethnic populations

	**DXS101**				**DXS7133**			
	Sinhala	SLT	INT	Moors	Sinhala	SLT	INT	Moors
PIC	0.7955	0.8251	0.8167	0.7976	0.6028	0.5630	0.5600	0.5968
He	0.8178	0.8437	0.8364	0.8204	0.6523	0.6028	0.6027	0.6558
PD_f	0.9445	0.9569	0.9534	0.9448	0.8296	0.8025	0.7995	0.8225
PD_m	0.8178	0.8437	0.8364	0.8204	0.6523	0.6028	0.6027	0.6558
MEC_{Kru}	0.6478	0.6895	0.6769	0.6488	0.4089	0.3773	0.3721	0.3939
$MEC_{Des\text{-}trio}$	0.7955	0.8251	0.8167	0.7976	0.6028	0.5630	0.5600	0.5968
$MEC_{Des\text{-}duo}$	0.6780	0.7166	0.7053	0.6803	0.4556	0.4139	0.4110	0.4496

Supplementary Table S13. Forensic parameters of 16 X-STR loci among the four ethnic populations.

	**HPRTB**				**DXS7423**			
	Sinhala	SLT	INT	Moors	Sinhala	SLT	INT	Moors
PIC	0.7078	0.6790	0.6713	0.6912	0.5466	0.5146	0.4956	0.5788
He	0.7487	0.7263	0.7179	0.7358	0.6220	0.5983	0.5840	0.6460
PD_f	0.8959	0.8778	0.8738	0.8856	0.7817	0.7549	0.7385	0.8074
PD_m	0.7487	0.7263	0.7179	0.7358	0.6220	0.5983	0.5840	0.6460
MEC_{Kru}	0.5212	0.4849	0.4774	0.5011	0.3432	0.3127	0.2958	0.3775
$MEC_{Des\text{-}trio}$	0.7078	0.6790	0.6713	0.6912	0.5466	0.5146	0.4956	0.5788
$MEC_{Des\text{-}duo}$	0.5698	0.5375	0.5288	0.5514	0.4020	0.3720	0.3548	0.4336

PIC: polymorphism information content, He: expected heterozygosity, PD female: power of discrimination in females, PD male: power of discrimination in males, MEC Krü: mean exclusion chance Kruger, MEC Des.trio: mean exclusion chance Desmaris trio. MEC Des.duo: mean exclusion chance Desmaris duo, SLT: Sri Lankan Tamil, INT: Indian Tamil.

Supplementary Table S14. Pairwise Fst values calculated for DXS10148

DXS10148	Sinhala	SL Tamil	IND Tamil	Moors	Bhil India	Bangladesh	Malaysia	Thailand	China
Sinhala		0.0527	0.0411	0.1054	0.2828	0.0774	0.0099	0.0077	0.0003
SL Tamil	0.0025		0.3100	0.7555	0.7544	0.7180	0.0359	0.0013	0.0024
IND Tamil	0.0031	0.0007		0.1046	0.2029	0.0403	0.0198	0.0030	0.0010
Moors	0.0021	-0.0017	0.0030		0.6308	0.3559	0.0339	0.0050	0.0021
Bhil India	0.0005	-0.0014	0.0015	-0.0010		0.5522	0.0066	0.0017	0.0008
Bangladesh	0.0017	-0.0011	0.0039	0.0004	-0.0005		0.0079	0.0045	0.0020
Malaysia	0.0027	0.0031	0.0044	0.0037	0.0048	0.0038		0.1572	0.0780
Thailand	0.0028	0.0067	0.0062	0.0057	0.0055	0.0042	0.0009		0.0608
China	0.0041	0.0058	0.0072	0.0067	0.0063	0.0041	0.0013	0.0013	
Japan	0.0041	0.0034	0.0056	0.0036	0.0029	0.0025	0.0029	0.0020	0.0035
Taiwan	0.0056	0.0071	0.0109	0.0092	0.0079	0.0048	0.0020	0.0015	0.0003
Germany	0.0074	0.0055	0.0136	0.0066	0.0072	0.0034	0.0076	0.0078	0.0054
Italy	0.0063	0.0027	0.0106	0.0059	0.0036	0.0011	0.0104	0.0107	0.0067
Sweden	0.0075	0.0045	0.0129	0.0067	0.0071	0.0037	0.0084	0.0087	0.0061
Denmark	0.0117	0.0106	0.0197	0.0118	0.0139	0.0074	0.0127	0.0119	0.0099
North Portugal	0.0031	0.0050	0.0095	0.0068	0.0047	0.0039	0.0037	0.0045	0.0015
Somalia	0.0285	0.0291	0.0350	0.0285	0.0288	0.0259	0.0273	0.0253	0.0280
Ivory Coast	0.0351	0.0355	0.0317	0.0362	0.0352	0.0352	0.0322	0.0335	0.0348

DXS10148	Japan	Taiwan	Germany	Italy	Sweden	Denmark	North Portugal	Somalia	Ivory Coast
Sinhala	0.0000	0.0000	0.0000	0.0061	0.0000	0.0000	0.0184	0.0000	0.0000
SL Tamil	0.0119	0.0001	0.0029	0.1352	0.0115	0.0011	0.0186	0.0000	0.0000
IND Tamil	0.0031	0.0000	0.0000	0.0048	0.0001	0.0000	0.0012	0.0000	0.0000
Moors	0.0179	0.0001	0.0022	0.0374	0.0041	0.0009	0.0091	0.0000	0.0000
Bhil India	0.0162	0.0001	0.0003	0.0851	0.0006	0.0000	0.0160	0.0000	0.0000
Bangladesh	0.0150	0.0019	0.0074	0.2620	0.0121	0.0036	0.0212	0.0000	0.0000
Malaysia	0.0035	0.0295	0.0000	0.0002	0.0000	0.0000	0.0143	0.0000	0.0000
Thailand	0.0110	0.0463	0.0000	0.0001	0.0000	0.0003	0.0041	0.0000	0.0000
China	0.0002	0.2916	0.0000	0.0043	0.0000	0.0001	0.0961	0.0000	0.0000
Japan		0.0002	0.0000	0.0000	0.0000	0.0000	0.0000	0.0000	0.0000
Taiwan	0.0031		0.0000	0.0009	0.0000	0.0000	0.0345	0.0000	0.0000
Germany	0.0092	0.0062		0.4381	0.7628	0.5719	0.1808	0.0000	0.0000
Italy	0.0101	0.0079	-0.0001		0.7143	0.2554	0.3292	0.0000	0.0000
Sweden	0.0102	0.0065	-0.0005	-0.0013		0.5249	0.2231	0.0000	0.0000
Denmark	0.0154	0.0109	-0.0005	0.0013	-0.0004		0.0657	0.0000	0.0000
North Portugal	0.0077	0.0025	0.0009	0.0007	0.0008	0.0034		0.0000	0.0000
Somalia	0.0234	0.0257	0.0324	0.0336	0.0344	0.0387	0.0304		0.0000
Ivory Coast	0.0305	0.0380	0.0425	0.0428	0.0464	0.0477	0.0384	0.0205	

Below diagonal: Pairwise Fst values; Above diagonal: P-values with shaded cells indicating P<0.0003 (after correction: 0.05/153)

Supplementary Table S14. Pairwise Fst values calculated for DXS10135

DXS10135	Sinhala	SLTamil	INDTamil	Moors	BhilIndia	Bangladesh	Malaysia	Thailand	China
Sinhala		0.5615	0.2306	0.3172	0.0062	0.3489	0.0000	0.0000	0.0000
SLTamil	-0.0003		0.2021	0.2893	0.0112	0.1040	0.0015	0.0018	0.0000
INDTamil	0.0009	0.0013		0.0271	0.0013	0.0439	0.0000	0.0000	0.0000
Moors	0.0005	0.0008	0.0045		0.0829	0.0724	0.0000	0.0000	0.0000
BhilIndia	0.0033	0.0040	0.0071	0.0025		0.0448	0.0000	0.0020	0.0000
Bangladesh	0.0003	0.0018	0.0030	0.0025	0.0024		0.0000	0.0000	0.0000
Malaysia	0.0059	0.0055	0.0119	0.0119	0.0074	0.0081		0.7831	0.0066
Thailand	0.0056	0.0046	0.0106	0.0093	0.0044	0.0071	-0.0007		0.0022
China	0.0083	0.0095	0.0169	0.0126	0.0086	0.0071	0.0026	0.0027	
Japan	0.0080	0.0080	0.0155	0.0120	0.0082	0.0088	0.0011	0.0015	0.0008
Taiwan	0.0082	0.0079	0.0161	0.0106	0.0089	0.0088	0.0025	0.0017	0.0007
Germany	0.0024	0.0014	0.0057	0.0052	0.0053	0.0044	0.0029	0.0042	0.0068
Italy	0.0038	0.0033	0.0100	0.0047	0.0084	0.0052	0.0057	0.0076	0.0069
Sweden	0.0026	0.0014	0.0062	0.0059	0.0054	0.0050	0.0023	0.0036	0.0075
Denmark	0.0014	0.0007	0.0064	0.0068	0.0056	0.0034	0.0005	0.0021	0.0030
NorthPortugal	0.0019	0.0024	0.0059	0.0042	0.0039	0.0033	0.0028	0.0037	0.0067
Somalia	0.0060	0.0057	0.0113	0.0069	0.0076	0.0064	0.0110	0.0113	0.0129
IvoryCoast	0.0232	0.0181	0.0275	0.0265	0.0209	0.0218	0.0143	0.0148	0.0159

DXS10135	Japan	Taiwan	Germany	Italy	Sweden	Denmark	North Portugal	Somalia	Ivory Coast
Sinhala	0.0000	0.0000	0.0002	0.0159	0.0012	0.1268	0.0382	0.0000	0.0000
SLTamil	0.0000	0.0000	0.0847	0.0567	0.0988	0.2890	0.0592	0.0006	0.0000
INDTamil	0.0000	0.0000	0.0006	0.0004	0.0004	0.0030	0.0011	0.0000	0.0000
Moors	0.0000	0.0000	0.0004	0.0300	0.0005	0.0022	0.0127	0.0006	0.0000
BhilIndia	0.0000	0.0000	0.0000	0.0007	0.0001	0.0036	0.0069	0.0000	0.0000
Bangladesh	0.0000	0.0000	0.0000	0.0096	0.0002	0.0227	0.0139	0.0002	0.0000
Malaysia	0.0762	0.0068	0.0003	0.0037	0.0088	0.3052	0.0172	0.0000	0.0000
Thailand	0.0199	0.0221	0.0000	0.0004	0.0000	0.0694	0.0023	0.0000	0.0000
China	0.0794	0.1099	0.0000	0.0014	0.0000	0.0211	0.0000	0.0000	0.0000
Japan		0.2134	0.0000	0.0001	0.0000	0.0298	0.0000	0.0000	0.0000
Taiwan	0.0003		0.0000	0.0005	0.0000	0.0022	0.0000	0.0000	0.0000
Germany	0.0062	0.0078		0.5007	0.8109	0.6346	0.7637	0.0000	0.0000
Italy	0.0075	0.0081	-0.0002		0.5114	0.4344	0.3608	0.0078	0.0000
Sweden	0.0065	0.0077	-0.0004	-0.0002		0.8600	0.6709	0.0000	0.0000
Denmark	0.0026	0.0048	-0.0005	0.0000	-0.0012		0.4763	0.0055	0.0000
NorthPortugal	0.0071	0.0091	-0.0006	0.0003	-0.0005	-0.0002		0.0003	0.0000
Somalia	0.0128	0.0135	0.0053	0.0050	0.0054	0.0044	0.0061		0.0000
IvoryCoast	0.0136	0.0171	0.0125	0.0145	0.0144	0.0121	0.0141	0.0113	

Below diagonal: Pairwise Fst values; Above diagonal: P-values with shaded cells indicating P<0.0003 (after correction: 0.05/153)

Supplementary Table S14. Pairwise Fst values calculated for DXS8378

DXS8378	Sinhala	SL Tamil	IND Tamil	Moors	Bhil India	Bangladesh	Malaysia	Thailand	China	Japan	Taiwan
Sinhala		0.6545	0.0384	0.4109	0.0963	0.0668	0.0000	0.0000	0.0000	0.0000	0.0000
SL Tamil	-0.0015		0.0920	0.5010	0.2241	0.2950	0.0000	0.0000	0.0000	0.0000	0.0000
IND Tamil	0.0063	0.0056		0.0371	0.0012	0.5947	0.0000	0.0000	0.0000	0.0000	0.0000
Moors	-0.0003	-0.0013	0.0105		0.6393	0.0801	0.0000	0.0000	0.0000	0.0000	0.0000
Bhil India	0.0031	0.0018	0.0213	-0.0021		0.0048	0.0000	0.0000	0.0000	0.0000	0.0000
Bangladesh	0.0036	0.0007	-0.0017	0.0058	0.0142		0.0000	0.0000	0.0000	0.0000	0.0000
Malaysia	0.0625	0.0657	0.0987	0.0412	0.0369	0.0875		0.8915	0.0252	0.0110	0.2741
Thailand	0.0604	0.0629	0.0977	0.0395	0.0346	0.0854	-0.0018		0.0066	0.0025	0.2764
China	0.1081	0.1143	0.1528	0.0831	0.0763	0.1389	0.0049	0.0069		0.8011	0.0282
Japan	0.1146	0.1207	0.1607	0.0889	0.0821	0.1456	0.0065	0.0081	-0.0009		0.0117
Taiwan	0.0787	0.0816	0.1215	0.0563	0.0479	0.1076	0.0005	0.0004	0.0035	0.0046	
Germany	0.0194	0.0175	0.0507	0.0119	0.0040	0.0384	0.0311	0.0272	0.0621	0.0659	0.0347
Italy	0.0014	-0.0018	0.0181	-0.0032	-0.0034	0.0092	0.0440	0.0406	0.0869	0.0920	0.0558
Sweden	0.0120	0.0102	0.0385	0.0049	-0.0001	0.0279	0.0304	0.0271	0.0641	0.0684	0.0370
Denmark	0.0094	0.0060	0.0356	0.0069	0.0011	0.0237	0.0515	0.0467	0.0943	0.0994	0.0589
North Portugal	0.0050	0.0021	0.0255	0.0001	-0.0024	0.0157	0.0411	0.0376	0.0813	0.0862	0.0509
Somalia	0.0291	0.0268	0.0659	0.0183	0.0084	0.0516	0.0238	0.0198	0.0520	0.0555	0.0248
Ivory Coast	0.0180	0.0139	0.0382	0.0262	0.0191	0.0287	0.1064	0.1010	0.1611	0.1687	0.1162
Pakistan	-0.0002	-0.0026	0.0091	-0.0013	0.0006	0.0039	0.0583	0.0558	0.1026	0.1086	0.0728
Brazil	0.0263	0.0251	0.0593	0.0136	0.0066	0.0470	0.0143	0.0115	0.0398	0.0431	0.0174
Brah. India	-0.0059	-0.0045	0.0046	-0.0070	-0.0039	0.0026	0.0469	0.0456	0.0932	0.1003	0.0635

DXS8378	Germany	Italy	Sweden	Denmark	North Portugal	Somalia	Ivory Coast	Pakistan	Brazil	Brah. India
Sinhala	0.0000	0.2460	0.0002	0.0129	0.0345	0.0000	0.0008	0.4438	0.0000	0.9220
SL Tamil	0.0000	0.5341	0.0072	0.0838	0.2023	0.0000	0.0124	0.9591	0.0001	0.6802
IND Tamil	0.0000	0.0113	0.0000	0.0000	0.0002	0.0000	0.0000	0.0175	0.0000	0.1973
Moors	0.0036	0.6921	0.0767	0.0734	0.3604	0.0030	0.0012	0.5705	0.0078	0.8866
BhilIndia	0.0601	0.7886	0.3853	0.2736	0.7791	0.0283	0.0017	0.2855	0.0347	0.6483
Bangladesh	0.0000	0.0434	0.0000	0.0001	0.0021	0.0000	0.0000	0.0561	0.0000	0.2534
Malaysia	0.0000	0.0000	0.0000	0.0000	0.0000	0.0003	0.0000	0.0000	0.0015	0.0004
Thailand	0.0000	0.0001	0.0000	0.0000	0.0000	0.0002	0.0000	0.0000	0.0016	0.0005
China	0.0000	0.0000	0.0000	0.0000	0.0000	0.0000	0.0000	0.0000	0.0000	0.0000
Japan	0.0000	0.0000	0.0000	0.0000	0.0000	0.0000	0.0000	0.0000	0.0000	0.0000
Taiwan	0.0000	0.0000	0.0000	0.0000	0.0000	0.0000	0.0000	0.0000	0.0000	0.0000
Germany		0.0828	0.4309	0.2601	0.0908	0.5392	0.0000	0.0000	0.1563	0.0513
Italy	0.0048		0.3674	0.4840	0.9489	0.0392	0.0157	0.6013	0.0402	0.5555
Sweden	-0.0002	0.0000		0.3746	0.5164	0.1965	0.0001	0.0013	0.1540	0.1729
Denmark	0.0010	-0.0014	-0.0001		0.6019	0.0985	0.0643	0.0549	0.0270	0.1758
North Portugal	0.0026	-0.0043	-0.0007	-0.0018		0.0445	0.0057	0.1921	0.0290	0.3795
Somalia	-0.0008	0.0100	0.0014	0.0052	0.0067		0.0000	0.0001	0.6091	0.0221
Ivory Coast	0.0234	0.0156	0.0222	0.0076	0.0157	0.0340		0.0016	0.0000	0.0253
Pakistan	0.0148	-0.0017	0.0082	0.0054	0.0014	0.0229	0.0153		0.0000	0.7297
Brazil	0.0014	0.0090	0.0017	0.0088	0.0067	-0.0014	0.0422	0.0211		0.0338
Brah. India	0.0119	-0.0034	0.0046	0.0056	-0.0003	0.0204	0.0197	-0.0043	0.0159	

Below diagonal: Pairwise Fst values; Above diagonal: P-values with shaded cells indicating P<0.0002 (after correction: 0.05/210)

Supplementary Table S14. Pairwise Fst values calculated for DXS7132

DXS7132	Sinhala	SL Tamil	IND Tamil	Moors	Bhil India	Bangladesh	Malaysia	Thailand	China	Japan
Sinhala		0.6205	0.1206	0.7337	0.2425	0.2279	0.0000	0.0000	0.0001	0.0000
SL Tamil	-0.0012		0.6256	0.4806	0.4174	0.8849	0.0002	0.0748	0.1585	0.0001
IND Tamil	0.0030	-0.0020		0.2233	0.4629	0.4402	0.0240	0.0888	0.1248	0.0138
Moors	-0.0019	-0.0009	0.0023		0.2206	0.4258	0.0000	0.0013	0.0026	0.0000
BhilIndia	0.0009	-0.0003	-0.0006	0.0018		0.0818	0.0004	0.0013	0.0028	0.0000
Bangladesh	0.0010	-0.0028	-0.0005	-0.0004	0.0040		0.0000	0.0453	0.0795	0.0000
Malaysia	0.0306	0.0205	0.0083	0.0305	0.0174	0.0235		0.0000	0.0000	0.0407
Thailand	0.0141	0.0041	0.0040	0.0157	0.0123	0.0044	0.0170		0.9033	0.0008
China	0.0106	0.0019	0.0029	0.0127	0.0096	0.0027	0.0184	-0.0013		0.0000
Japan	0.0281	0.0161	0.0080	0.0278	0.0190	0.0170	0.0030	0.0073	0.0097	
Taiwan	0.0114	0.0036	-0.0008	0.0103	0.0066	0.0042	0.0062	0.0034	0.0039	0.0025
Germany	0.0011	-0.0023	-0.0010	0.0020	0.0000	0.0003	0.0182	0.0058	0.0037	0.0157
Italy	0.0052	0.0009	-0.0007	0.0005	0.0076	-0.0015	0.0189	0.0082	0.0075	0.0135
Sweden	0.0026	0.0004	-0.0012	0.0020	-0.0016	0.0037	0.0133	0.0114	0.0094	0.0150
Denmark	-0.0025	-0.0020	0.0011	-0.0019	-0.0021	0.0016	0.0250	0.0138	0.0102	0.0249
North Portugal	0.0017	-0.0015	0.0048	0.0026	0.0063	-0.0011	0.0349	0.0059	0.0036	0.0252
Somalia	0.0607	0.0739	0.0801	0.0465	0.0779	0.0675	0.1267	0.1110	0.1056	0.1220
Ivory Coast	0.0025	-0.0029	-0.0040	0.0037	-0.0005	-0.0005	0.0109	0.0017	0.0003	0.0094
Pakistan	-0.0016	-0.0012	0.0022	-0.0023	0.0001	0.0012	0.0282	0.0142	0.0109	0.0265
Brah. India	0.0057	0.0012	-0.0044	-0.0001	0.0046	-0.0006	0.0090	0.0091	0.0087	0.0087

DXS7132	Taiwan	Germany	Italy	Sweden	Denmark	North Portugal	Somalia	Ivory Coast	Pakistan	Brah. India
Sinhala	0.0000	0.1336	0.0656	0.0450	0.9120	0.1591	0.0000	0.1417	0.9848	0.1378
SL Tamil	0.0671	0.9584	0.3036	0.3201	0.6512	0.6184	0.0000	0.7747	0.6123	0.3280
IND Tamil	0.5290	0.5620	0.4501	0.6117	0.2891	0.0897	0.0000	0.9198	0.1710	0.6950
Moors	0.0061	0.1653	0.3461	0.1739	0.5987	0.1708	0.0000	0.1520	0.8203	0.3995
BhilIndia	0.0098	0.3788	0.0469	0.8025	0.7089	0.0360	0.0000	0.4460	0.3674	0.1846
Bangladesh	0.0301	0.3309	0.5732	0.0515	0.2268	0.5804	0.0000	0.4390	0.2089	0.4139
Malaysia	0.0044	0.0000	0.0012	0.0000	0.0001	0.0000	0.0000	0.0090	0.0000	0.0816
Thailand	0.0267	0.0035	0.0265	0.0000	0.0032	0.0270	0.0000	0.2017	0.0000	0.0791
China	0.0087	0.0079	0.0285	0.0001	0.0052	0.0583	0.0000	0.3311	0.0000	0.0784
Japan	0.0265	0.0000	0.0023	0.0000	0.0000	0.0000	0.0000	0.0065	0.0000	0.0694
Taiwan		0.0032	0.1854	0.0085	0.0052	0.0014	0.0000	0.2735	0.0001	0.4770
Germany	0.0044		0.0836	0.1706	0.5009	0.1144	0.0000	0.8312	0.1587	0.2083
Italy	0.0020	0.0041		0.0748	0.1018	0.0907	0.0000	0.2035	0.0874	0.9130
Sweden	0.0042	0.0008	0.0047		0.4733	0.0047	0.0000	0.4187	0.1138	0.3345
Denmark	0.0094	-0.0006	0.0060	-0.0005		0.1448	0.0000	0.3412	0.9503	0.1929
North Portugal	0.0105	0.0021	0.0052	0.0083	0.0031		0.0000	0.1715	0.0959	0.0765
Somalia	0.0903	0.0795	0.0563	0.0739	0.0667	0.0754		0.0000	0.0000	0.0000
Ivory Coast	0.0008	-0.0020	0.0028	-0.0003	0.0005	0.0026	0.0903		0.1676	0.3626
Pakistan	0.0103	0.0009	0.0046	0.0014	-0.0027	0.0026	0.0598	0.0022		0.1754
Brah. India	-0.0011	0.0033	-0.0075	0.0007	0.0051	0.0095	0.0588	0.0003	0.0042	

Below diagonal: Pairwise Fst values; Above diagonal: P-values with shaded cells indicating P<0.0003 (after correction: 0.05/190)

Supplementary Table S14. Pairwise Fst values calculated for DXS10079

DXS10079	Sinhala	SL Tamil	IND Tamil	Moors	Bhil India	Bangladesh	Malaysia	Thailand	China
Sinhala		0.2523	0.7297	0.4054	0.5135	0.5676	0.0000	0.0270	0.0000
SL Tamil	0.0008		0.4054	0.0901	0.0991	0.2162	0.1171	0.1171	0.1261
IND Tamil	-0.0014	0.0008		0.1622	0.4775	0.7387	0.0451	0.0451	0.0000
Moors	0.0001	0.0039	0.0022		0.4324	0.4324	0.0451	0.1441	0.0000
Bhil India	-0.0004	0.0030	-0.0006	-0.0003		0.6667	0.0000	0.0360	0.0000
Bangladesh	-0.0004	0.0013	-0.0019	-0.0003	-0.0010		0.0270	0.2072	0.0000
Malaysia	0.0056	0.0022	0.0061	0.0029	0.0074	0.0047		0.8288	0.0000
Thailand	0.0039	0.0019	0.0038	0.0019	0.0051	0.0016	-0.0012		0.0000
China	0.0068	0.0012	0.0080	0.0109	0.0058	0.0082	0.0060	0.0066	
Japan	0.0060	0.0026	0.0072	0.0113	0.0073	0.0112	0.0088	0.0106	0.0017
Taiwan	0.0014	0.0005	-0.0018	0.0061	0.0030	0.0026	0.0077	0.0072	0.0071
Germany	-0.0004	0.0022	-0.0026	0.0016	0.0009	-0.0004	0.0060	0.0039	0.0094
Italy	0.0051	0.0018	0.0048	0.0073	0.0030	0.0005	0.0116	0.0072	0.0079
Sweden	-0.0011	0.0004	-0.0017	0.0013	-0.0001	-0.0008	0.0060	0.0036	0.0065
Denmark	0.0001	-0.0010	0.0032	-0.0005	0.0005	0.0021	0.0047	0.0042	0.0037
North Portugal	-0.0011	0.0005	-0.0032	0.0029	-0.0009	-0.0011	0.0087	0.0060	0.0071
Somalia	0.0000	0.0029	0.0007	-0.0018	-0.0025	-0.0019	0.0052	0.0024	0.0071
Ivory Coast	-0.0004	0.0017	0.0015	0.0027	-0.0010	0.0038	0.0084	0.0083	0.0030

DXS10079	Japan	Taiwan	Germany	Italy	Sweden	Denmark	North Portugal	Somalia	Ivory Coast
Sinhala	0.0000	0.0991	0.6126	0.0901	0.9369	0.4324	0.7478	0.4144	0.4234
SL Tamil	0.0901	0.2793	0.0811	0.2523	0.3604	0.5405	0.2793	0.1351	0.2613
IND Tamil	0.0090	0.8198	0.9910	0.1171	0.7838	0.0991	0.9189	0.3153	0.2883
Moors	0.0090	0.0180	0.1532	0.0541	0.2072	0.4775	0.1171	0.7117	0.1441
Bhil India	0.0000	0.0270	0.2162	0.1441	0.4685	0.2973	0.4414	0.9009	0.4685
Bangladesh	0.0000	0.0631	0.6216	0.3063	0.7207	0.1351	0.7387	0.8198	0.0631
Malaysia	0.0000	0.0000	0.0000	0.0090	0.0000	0.0541	0.0000	0.0090	0.0000
Thailand	0.0000	0.0000	0.0090	0.0180	0.0180	0.0541	0.0090	0.1261	0.0000
China	0.0270	0.0000	0.0000	0.0000	0.0000	0.0180	0.0000	0.0000	0.1081
Japan		0.0000	0.0000	0.0000	0.0090	0.0541	0.0000	0.0000	0.2973
Taiwan	0.0052		0.1081	0.0270	0.0901	0.0541	0.4955	0.0000	0.1351
Germany	0.0085	0.0011		0.0000	0.6487	0.0360	0.7117	0.1802	0.0631
Italy	0.0150	0.0079	0.0066		0.1622	0.2072	0.2162	0.1802	0.0180
Sweden	0.0065	0.0017	-0.0005	0.0033		0.2883	0.9099	0.3784	0.3153
Denmark	0.0032	0.0039	0.0031	0.0038	0.0004		0.2252	0.3964	0.5045
North Portugal	0.0068	-0.0003	-0.0009	0.0022	-0.0016	0.0016		0.2342	0.3333
Somalia	0.0103	0.0051	0.0014	0.0016	0.0002	0.0001	0.0008		0.2703
Ivory Coast	0.0006	0.0020	0.0026	0.0085	0.0008	-0.0010	0.0002	0.0020	

Below diagonal: Pairwise Fst values; Above diagonal: P-values with shaded cells indicating P<0.0003 (after correction: 0.05/153)

Supplementary Table S14. Pairwise Fst values calculated for DXS10074

DXS10074	Sinhala	SL Tamil	IND Tamil	Moors	Bhil India	Bangladesh	Malaysia	Thailand	China
Sinhala		0.6937	0.3964	0.4865	0.1712	0.4054	0.0000	0.0000	0.0000
SL Tamil	-0.0013		0.6667	0.5135	0.1802	0.4775	0.0000	0.0000	0.0270
IND Tamil	-0.0005	-0.0016		0.6216	0.1892	0.9550	0.0090	0.1081	0.1712
Moors	-0.0005	-0.0009	-0.0019		0.9730	0.2883	0.0000	0.0631	0.1171
Bhil India	0.0018	0.0016	0.0018	-0.0035		0.0360	0.0000	0.0090	0.0180
Bangladesh	-0.0002	-0.0004	-0.0033	0.0008	0.0052		0.0000	0.0090	0.0180
Malaysia	0.0214	0.0170	0.0092	0.0159	0.0205	0.0142		0.1351	0.0000
Thailand	0.0102	0.0066	0.0031	0.0068	0.0096	0.0071	0.0014		0.0360
China	0.0055	0.0062	0.0021	0.0038	0.0053	0.0052	0.0090	0.0024	
Japan	0.0085	0.0075	0.0044	0.0048	0.0064	0.0081	0.0062	0.0009	0.0003
Taiwan	0.0072	0.0077	0.0018	0.0053	0.0077	0.0050	0.0065	0.0013	-0.0007
Germany	0.0099	0.0089	0.0079	0.0042	0.0069	0.0091	0.0320	0.0248	0.0232
Italy	0.0227	0.0197	0.0192	0.0110	0.0118	0.0233	0.0388	0.0327	0.0341
Sweden	0.0089	0.0094	0.0085	0.0030	0.0048	0.0100	0.0321	0.0241	0.0206
Denmark	0.0053	0.0070	0.0046	0.0037	0.0075	0.0040	0.0341	0.0248	0.0205
North Portugal	0.0145	0.0119	0.0110	0.0061	0.0084	0.0142	0.0256	0.0210	0.0232
Somalia	0.0617	0.0576	0.0560	0.0477	0.0520	0.0590	0.0803	0.0768	0.0832
Ivory Coast	0.0649	0.0574	0.0650	0.0605	0.0638	0.0678	0.1020	0.0888	0.0960

DXS10074	Japan	Taiwan	Germany	Italy	Sweden	Denmark	North Portugal	Somalia	Ivory Coast
Sinhala	0.0000	0.0000	0.0000	0.0000	0.0000	0.0360	0.0000	0.0000	0.0000
SL Tamil	0.0000	0.0180	0.0000	0.0000	0.0000	0.0541	0.0000	0.0000	0.0000
IND Tamil	0.0541	0.1712	0.0000	0.0000	0.0000	0.0721	0.0090	0.0000	0.0000
Moors	0.0451	0.0360	0.0360	0.0180	0.1171	0.0811	0.0360	0.0000	0.0000
Bhil India	0.0000	0.0090	0.0000	0.0000	0.0180	0.0090	0.0090	0.0000	0.0000
Bangladesh	0.0000	0.0000	0.0000	0.0000	0.0000	0.0631	0.0000	0.0000	0.0000
Malaysia	0.0000	0.0000	0.0000	0.0000	0.0000	0.0000	0.0000	0.0000	0.0000
Thailand	0.1892	0.0721	0.0000	0.0000	0.0000	0.0000	0.0000	0.0000	0.0000
China	0.2703	0.7387	0.0000	0.0000	0.0000	0.0000	0.0000	0.0000	0.0000
Japan		0.1081	0.0000	0.0000	0.0000	0.0000	0.0000	0.0000	0.0000
Taiwan	0.0010		0.0000	0.0000	0.0000	0.0000	0.0000	0.0000	0.0000
Germany	0.0249	0.0241		0.0991	0.3514	0.4054	0.0721	0.0000	0.0000
Italy	0.0341	0.0355	0.0026		0.1982	0.0090	0.7027	0.0000	0.0000
Sweden	0.0226	0.0222	0.0000	0.0017		0.3333	0.1802	0.0000	0.0000
Denmark	0.0249	0.0209	-0.0001	0.0090	0.0003		0.0451	0.0000	0.0000
North Portugal	0.0229	0.0239	0.0028	-0.0017	0.0011	0.0063		0.0000	0.0000
Somalia	0.0814	0.0837	0.0264	0.0172	0.0268	0.0321	0.0234		0.0000
Ivory Coast	0.0947	0.0981	0.0497	0.0555	0.0526	0.0507	0.0547	0.0376	

Below diagonal: Pairwise Fst values; Above diagonal: P-values with shaded cells indicating P<0.0003 (after correction: 0.05/153)

Supplementary Table S14. Pairwise Fst values calculated for HPRTB

HPRTB	Sinhala	SL Tamil	IND Tamil	Moors	Bhil India	Bangladesh	Malaysia	Thailand	China	Japan	Taiwan
Sinhala		0.4768	0.1409	0.1498	0.0147	0.4415	0.0065	0.1059	0.1178	0.0000	0.0255
SL Tamil	-0.0006		0.3823	0.3759	0.0527	0.7840	0.0266	0.4003	0.7144	0.0096	0.6726
IND Tamil	0.0026	-0.0001		0.0149	0.1134	0.0832	0.0045	0.0157	0.3272	0.0034	0.0286
Moors	0.0024	0.0000	0.0126		0.0027	0.6388	0.0124	0.4838	0.0424	0.0029	0.3671
Bhil India	0.0064	0.0062	0.0042	0.0168		0.0316	0.0994	0.0034	0.0922	0.0967	0.0081
Bangladesh	-0.0003	-0.0023	0.0050	-0.0017	0.0064		0.0729	0.7280	0.3137	0.0048	0.7606
Malaysia	0.0061	0.0069	0.0124	0.0099	0.0031	0.0035		0.0571	0.0248	0.0017	0.0084
Thailand	0.0018	-0.0001	0.0086	-0.0006	0.0105	-0.0014	0.0033		0.0908	0.0000	0.2473
China	0.0014	-0.0015	0.0005	0.0056	0.0029	0.0003	0.0040	0.0019		0.0004	0.1231
Japan	0.0122	0.0086	0.0137	0.0143	0.0027	0.0078	0.0089	0.0134	0.0079		0.0014
Taiwan	0.0032	-0.0014	0.0068	0.0000	0.0077	-0.0014	0.0058	0.0006	0.0013	0.0063	
Germany	0.0124	0.0116	0.0290	0.0016	0.0249	0.0057	0.0104	0.0042	0.0155	0.0217	0.0080
Italy	0.0095	0.0084	0.0264	-0.0015	0.0205	0.0027	0.0083	0.0026	0.0126	0.0148	0.0041
Sweden	0.0095	0.0075	0.0234	-0.0003	0.0202	0.0028	0.0081	0.0019	0.0112	0.0173	0.0044
Denmark	0.0243	0.0231	0.0474	0.0063	0.0401	0.0152	0.0251	0.0157	0.0300	0.0280	0.0162
North Portugal	0.0214	0.0202	0.0404	0.0079	0.0382	0.0134	0.0163	0.0082	0.0239	0.0377	0.0162
Somalia	0.0111	0.0169	0.0215	0.0154	0.0208	0.0129	0.0142	0.0131	0.0184	0.0332	0.0206
Ivory Coast	0.0059	0.0076	0.0187	0.0014	0.0234	0.0046	0.0146	0.0046	0.0130	0.0276	0.0096
Pakistan	0.0020	-0.0007	0.0071	0.0004	0.0060	-0.0018	0.0021	-0.0009	0.0006	0.0070	-0.0009
Brazil	0.0046	0.0035	0.0169	-0.0026	0.0192	0.0007	0.0085	-0.0004	0.0078	0.0193	0.0031
Brah. India	0.0096	0.0032	0.0052	0.0134	-0.0030	0.0048	0.0046	0.0092	0.0011	-0.0043	0.0020

HPRTB	Germany	Italy	Sweden	Denmark	North Portugal	Somalia	Ivory Coast	Pakistan	Brazil	Brah. India
Sinhala	0.0000	0.0167	0.0000	0.0000	0.0000	0.0009	0.0324	0.0802	0.0199	0.0649
SL Tamil	0.0017	0.0484	0.0147	0.0003	0.0005	0.0005	0.0406	0.5205	0.0959	0.2390
IND Tamil	0.0000	0.0013	0.0002	0.0000	0.0000	0.0000	0.0022	0.0264	0.0009	0.1759
Moors	0.1912	0.5200	0.4185	0.0734	0.0325	0.0016	0.2730	0.3228	0.8396	0.0612
Bhil India	0.0000	0.0023	0.0000	0.0000	0.0000	0.0002	0.0004	0.0234	0.0003	0.5926
Bangladesh	0.0091	0.1744	0.0771	0.0027	0.0012	0.0009	0.0782	0.8853	0.2845	0.1779
Malaysia	0.0001	0.0319	0.0018	0.0000	0.0002	0.0005	0.0019	0.1060	0.0050	0.1676
Thailand	0.0093	0.1685	0.0915	0.0012	0.0065	0.0002	0.0632	0.6696	0.4760	0.0746
China	0.0000	0.0046	0.0000	0.0000	0.0000	0.0000	0.0021	0.2326	0.0021	0.3105
Japan	0.0000	0.0051	0.0000	0.0000	0.0000	0.0000	0.0000	0.0020	0.0000	0.7711
Taiwan	0.0000	0.0995	0.0098	0.0010	0.0001	0.0000	0.0081	0.7371	0.0485	0.2629
Germany		0.8628	0.7917	0.0891	0.1824	0.0000	0.0383	0.0014	0.2811	0.0061
Italy	-0.0025		0.9083	0.5693	0.2113	0.0011	0.1091	0.1410	0.5339	0.0444
Sweden	-0.0007	-0.0029		0.0710	0.1127	0.0000	0.0521	0.0352	0.5587	0.0188
Denmark	0.0030	-0.0018	0.0039		0.0258	0.0000	0.0089	0.0008	0.0269	0.0047
North Portugal	0.0012	0.0022	0.0022	0.0085		0.0000	0.0148	0.0005	0.0751	0.0009
Somalia	0.0168	0.0180	0.0168	0.0313	0.0222		0.1635	0.0001	0.0003	0.0014
Ivory Coast	0.0049	0.0048	0.0046	0.0128	0.0097	0.0024		0.0199	0.2751	0.0054
Pakistan	0.0060	0.0031	0.0030	0.0162	0.0128	0.0151	0.0073		0.1181	0.2300
Brazil	0.0004	-0.0011	-0.0006	0.0074	0.0036	0.0130	0.0009	0.0019		0.0198
Brah. India	0.0222	0.0161	0.0163	0.0332	0.0354	0.0305	0.0262	0.0029	0.0175	

Below diagonal: Pairwise Fst values; Above diagonal: P-values with shaded cells indicating P<0.0002 (after correction: 0.05/210)

Supplementary Table S14. Pairwise Fst values calculated for DXS7423

DXS7423	Sinhala	SL Tamil	IND Tamil	Moors	Bhil India	Bangladesh	Malaysia	Thailand	China	Japan
Sinhala		0.6385	0.3254	0.5570	0.4598	0.5423	0.0284	0.3760	0.0000	0.0000
SL Tamil	-0.0017		0.8434	0.3492	0.4840	0.2243	0.0682	0.9032	0.0000	0.0000
IND Tamil	0.0002	-0.0039		0.2447	0.3421	0.1077	0.0588	0.5396	0.0000	0.0000
Moors	-0.0014	0.0000	0.0018		0.2554	0.5774	0.0285	0.2337	0.0000	0.0000
Bhil India	-0.0007	-0.0013	-0.0001	0.0013		0.2705	0.4275	0.2008	0.0000	0.0000
Bangladesh	-0.0010	0.0016	0.0052	-0.0018	0.0010		0.0362	0.1159	0.0000	0.0000
Malaysia	0.0060	0.0063	0.0078	0.0102	-0.0007	0.0070		0.0081	0.0000	0.0000
Thailand	-0.0002	-0.0027	-0.0015	0.0013	0.0016	0.0029	0.0104		0.0000	0.0000
China	0.0477	0.0386	0.0371	0.0496	0.0614	0.0583	0.0843	0.0340		0.4793
Japan	0.0522	0.0425	0.0406	0.0563	0.0666	0.0638	0.0892	0.0377	-0.0004	
Taiwan	0.0443	0.0345	0.0319	0.0484	0.0566	0.0561	0.0779	0.0307	-0.0002	-0.0006
Germany	0.0168	0.0212	0.0246	0.0073	0.0263	0.0124	0.0405	0.0208	0.0524	0.0596
Italy	0.0098	0.0092	0.0123	0.0047	0.0195	0.0106	0.0374	0.0070	0.0215	0.0282
Sweden	0.0156	0.0196	0.0233	0.0065	0.0253	0.0114	0.0403	0.0191	0.0500	0.0574
Denmark	0.0295	0.0330	0.0359	0.0165	0.0434	0.0253	0.0648	0.0318	0.0491	0.0576
North Portugal	0.0212	0.0260	0.0316	0.0117	0.0331	0.0151	0.0504	0.0241	0.0528	0.0608
Somalia	0.0030	0.0044	0.0051	-0.0019	0.0033	0.0036	0.0110	0.0069	0.0609	0.0694
Ivory Coast	0.0135	0.0169	0.0159	0.0068	0.0103	0.0127	0.0135	0.0222	0.0965	0.1053
Brazil	0.0185	0.0195	0.0238	0.0124	0.0310	0.0165	0.0497	0.0167	0.0276	0.0332

DXS7423	Taiwan	Germany	Italy	Sweden	Denmark	North Portugal	Somalia	Ivory Coast	Brazil
Sinhala	0.0000	0.0000	0.0289	0.0000	0.0001	0.0000	0.1146	0.0065	0.0001
SL Tamil	0.0000	0.0000	0.0647	0.0002	0.0002	0.0005	0.1079	0.0135	0.0015
IND Tamil	0.0003	0.0002	0.0402	0.0005	0.0003	0.0000	0.1009	0.0182	0.0010
Moors	0.0000	0.0195	0.1330	0.0400	0.0075	0.0146	0.6131	0.0791	0.0118
Bhil India	0.0000	0.0000	0.0097	0.0000	0.0000	0.0000	0.1346	0.0378	0.0000
Bangladesh	0.0000	0.0005	0.0329	0.0011	0.0001	0.0035	0.1086	0.0150	0.0007
Malaysia	0.0000	0.0000	0.0004	0.0000	0.0000	0.0000	0.0127	0.0131	0.0000
Thailand	0.0000	0.0000	0.0658	0.0000	0.0001	0.0000	0.0316	0.0014	0.0005
China	0.3791	0.0000	0.0026	0.0000	0.0000	0.0000	0.0000	0.0000	0.0000
Japan	0.5239	0.0000	0.0006	0.0000	0.0000	0.0000	0.0000	0.0000	0.0000
Taiwan		0.0000	0.0022	0.0000	0.0000	0.0000	0.0000	0.0000	0.0000
Germany	0.0554		0.0467	0.9780	0.3223	0.3220	0.0004	0.0000	0.0082
Italy	0.0250	0.0061		0.1053	0.0900	0.1300	0.0281	0.0010	0.5605
Sweden	0.0532	-0.0011	0.0040		0.3589	0.4863	0.0016	0.0000	0.0427
Denmark	0.0553	0.0004	0.0065	0.0001		0.3194	0.0010	0.0000	0.1105
North Portugal	0.0581	0.0003	0.0042	-0.0005	0.0006		0.0006	0.0000	0.2310
Somalia	0.0597	0.0126	0.0116	0.0123	0.0234	0.0206		0.1821	0.0002
Ivory Coast	0.0931	0.0247	0.0349	0.0258	0.0398	0.0382	0.0026		0.0000
Brazil	0.0319	0.0056	-0.0016	0.0038	0.0037	0.0012	0.0226	0.0462	

Below diagonal: Pairwise Fst values; Above diagonal: P-values with shaded cells indicating P<0.0003 (after correction: 0.05/171)

Supplementary Table S14. Pairwise Fst values calculated for DXS9902, DXS10075, DXS7133 and DXS6801

DXS9902	Sinhala	SL Tamil	IND Tamil	Moors	Pakistan	China	Taiwan	Germany	Italy
Sinhala		0.7704	0.0195	0.1041	0.1445	0.0000	0.0002	0.0044	0.1578
SL Tamil	-0.0017		0.0424	0.0748	0.1071	0.0000	0.0000	0.0045	0.0743
IND Tamil	0.0074	0.0082		0.0051	0.0367	0.0000	0.0000	0.0458	0.0333
Moors	0.0035	0.0059	0.0175		0.4719	0.0006	0.0029	0.1233	0.7552
Pakistan	0.0013	0.0031	0.0065	-0.0006		0.0000	0.0000	0.2251	0.7319
China	0.0159	0.0249	0.0272	0.0177	0.0172		0.3305	0.0001	0.0151
Taiwan	0.0212	0.0308	0.0415	0.0186	0.0228	0.0002		0.0000	0.0096
Germany	0.0068	0.0118	0.0063	0.0031	0.0008	0.0128	0.0199		0.6317
Italy	0.0025	0.0064	0.0107	-0.0033	-0.0021	0.0105	0.0143	-0.0017	

Below diagonal: Pairwise Fst values; Above diagonal: P-values with shaded cells indicating P<0.0014 (after correction:0.05/36)

DXS10075	Sinhala	SL Tamil	IND Tamil	Moors	China	Japan	Germany	Italy
Sinhala		0.6633	0.8709	0.2964	0.2727	0.0001	0.0316	0.7816
SL Tamil	-0.0014		0.7865	0.1857	0.4145	0.0001	0.0335	0.6875
IND Tamil	-0.0024	-0.0029		0.6188	0.2793	0.0037	0.1040	0.6985
Moors	0.0006	0.0027	-0.002		0.0432	0.0354	0.1888	0.3986
China	0.0004	-0.0004	0.0008	0.0061		0.0000	0.0003	0.3787
Japan	0.0121	0.0188	0.0133	0.0061	0.0184		0.0179	0.0215
Germany	0.0044	0.0069	0.0039	0.0019	0.0119	0.0048		0.3376
Italy	-0.0025	-0.0029	-0.0030	-0.0003	-0.0001	0.0094	0.0004	

Below diagonal: Pairwise Fst values; Above diagonal: P-values with shaded cells indicating P<0.0018 (after correction:0.05/28)

DXS7133	Sinhala	SL Tamil	IND Tamil	Moors	Brahmin India	China	Japan	Germany	Italy	Brazil
Sinhala		0.0293	0.0375	0.3710	0.8680	0.0000	0.0000	0.0107	0.0572	0.0078
SL Tamil	0.0068		0.8652	0.0076	0.2624	0.0000	0.0000	0.0000	0.0007	0.0000
IND Tamil	0.0067	-0.0034		0.0196	0.2455	0.0000	0.0000	0.0003	0.0020	0.0001
Moors	-0.0001	0.0166	0.0139		0.4098	0.0000	0.0000	0.3026	0.5295	0.4122
Brahmin India	-0.0054	0.0022	0.0030	-0.0010		0.0000	0.0000	0.2465	0.2228	0.1650
China	0.1254	0.0784	0.082	0.1770	0.1475		0.0000	0.0000	0.0000	0.0000
Japan	0.0805	0.0415	0.0392	0.1052	0.0946	0.0325		0.0000	0.0000	0.0000
Germany	0.0069	0.0309	0.0276	0.0006	0.0026	0.1868	0.1313		0.8370	0.3392
Italy	0.0063	0.0327	0.0288	-0.0019	0.0039	0.2186	0.1421	-0.0033		0.9324
Brazil	0.0074	0.0308	0.0265	-0.0004	0.0052	0.1935	0.1270	0.0001	-0.0037	

Below diagonal: Pairwise Fst values; Above diagonal: P-values with shaded cells indicating P<0.0011 (after correction:0.05/45)

DXS6801	Sinhala	SL Tamil	IND Tamil	Moors	Pakistan	China	Germany	Italy	Ivory Coast	Brazil
Sinhala		0.5928	0.0653	0.7843	0.1065	0.0000	0.0000	0.0000	0.0000	0.0000
SL Tamil	-0.0012		0.0401	0.3427	0.0877	0.0074	0.0000	0.0021	0.0000	0.0049
IND Tamil	0.0051	0.0087		0.4087	0.0072	0.0000	0.0000	0.0000	0.0000	0.0000
Moors	-0.0023	0.0003	-0.0004		0.2934	0.0000	0.0000	0.0001	0.0000	0.0000
Pakistan	0.0025	0.0045	0.0138	0.0008		0.0000	0.0000	0.0009	0.0000	0.0042
China	0.0184	0.0103	0.0492	0.0279	0.0242		0.0000	0.0347	0.0000	0.0068
Germany	0.0280	0.0201	0.0621	0.0357	0.0206	0.0093		0.2354	0.0000	0.3538
Italy	0.0276	0.0211	0.0646	0.0366	0.0212	0.0058	0.0008		0.0000	0.5010
Ivory Coast	0.0749	0.0677	0.0741	0.0677	0.0591	0.1073	0.0752	0.0955		0.0000
Brazil	0.0181	0.0128	0.0481	0.0240	0.0109	0.0072	0.0000	-0.0009	0.0732	

Below diagonal: Pairwise Fst values; Above diagonal: P-values with shaded cells indicating P<0.0018 (after correction:0.05/28)

Supplementary Table S14. Pairwise Fst values calculated for DXS6809 and DXS6789

DXS6809	Sinhala	SL Tamil	IND Tamil	Moors	Brahmin India	Pakistan	China	Japan	Taiwan	Germany	Italy	Brazil
Sinhala		0.5138	0.3993	0.6721	0.6298	0.1963	0.0000	0.0355	0.0016	0.0012	0.5953	0.0099
SL Tamil	-0.0004		0.4023	0.3673	0.2681	0.1968	0.0052	0.0270	0.0649	0.0080	0.1338	0.0269
IND Tamil	0.0000	-0.0000		0.2611	0.5043	0.2843	0.0361	0.3262	0.1948	0.1936	0.6266	0.2316
Moors	-0.0012	0.0003	0.0014		0.8702	0.9119	0.0896	0.5279	0.0568	0.0959	0.5670	0.2893
Brahmin India	-0.0023	0.0021	-0.0011	-0.0057		0.5612	0.1445	0.6947	0.1358	0.2911	0.7917	0.3676
Pakistan	0.0010	0.0016	0.0009	-0.0028	-0.0019		0.1853	0.4006	0.0718	0.3407	0.4292	0.7164
China	0.0074	0.0067	0.0043	0.0027	0.0041	0.0010		0.0450	0.3960	0.0128	0.0090	0.6685
Japan	0.0023	0.0046	0.0004	-0.0006	-0.0029	-0.0000	0.0017		0.0621	0.0681	0.5591	0.3272
Taiwan	0.0071	0.0040	0.0018	0.0046	0.0051	0.0030	0.0000	0.0024		0.0209	0.0276	0.2097
Germany	0.0045	0.0058	0.0013	0.0024	0.0013	0.0002	0.0020	0.0012	0.0031		0.2548	0.7598
Italy	-0.0008	0.0027	-0.0014	-0.0011	-0.0043	-0.0002	0.0055	-0.0006	0.0054	0.0007		0.1730
Brazil	0.0044	0.0052	0.0012	0.0007	0.0003	-0.0012	-0.0006	0.0003	0.0010	-0.0007	0.0017	

Below diagonal: Pairwise Fst values

Above diagonal: P-values P<0.0008 (after correction: 0.05/66) shaded

DXS6789	Sinhala	SL Tamil	IND Tamil	Moors	Brahmin India	Pakistan	China	Japan	Taiwan	Germany	Italy	Ivory Coast	Brazil
Sinhala		0.0492	0.1260	0.3671	0.8269	0.0010	0.0000	0.0000	0.0000	0.0000	0.0000	0.0000	0.0000
SL Tamil	0.0038		0.4643	0.8188	0.2159	0.0000	0.0000	0.0000	0.0000	0.0000	0.0000	0.0030	0.0000
IND Tamil	0.0025	-0.0005		0.8455	0.5921	0.0000	0.0000	0.0000	0.0000	0.0000	0.0000	0.0015	0.0000
Moors	0.0001	-0.0026	-0.0029		0.6125	0.0001	0.0000	0.0000	0.0000	0.0000	0.0000	0.0114	0.0000
Brahmin India	-0.0041	0.0034	-0.0026	-0.0027		0.1806	0.0000	0.0000	0.0001	0.0003	0.0012	0.0137	0.0042
Pakistan	0.0089	0.0297	0.0257	0.0208	0.0042		0.0000	0.0000	0.0000	0.0005	0.0112	0.0000	0.0341
China	0.0541	0.0409	0.0551	0.044	0.0606	0.0838		0.0057	0.0766	0.0000	0.0000	0.0000	0.0000
Japan	0.0478	0.0380	0.0518	0.0396	0.0518	0.0713	0.0036		0.3571	0.0000	0.0000	0.0000	0.0000
Taiwan	0.0364	0.0264	0.0382	0.0276	0.0407	0.0627	0.0022	0.0001		0.0000	0.0000	0.0000	0.0000
Germany	0.0392	0.0714	0.0673	0.0575	0.0345	0.0108	0.1174	0.0959	0.0917		0.3928	0.0000	0.0816
Italy	0.0421	0.0784	0.0726	0.0639	0.0381	0.0098	0.1244	0.1049	0.1005	-0.0001		0.0000	0.4896
Ivory Coast	0.0211	0.0128	0.0159	0.0101	0.0172	0.0460	0.0547	0.0391	0.0307	0.0710	0.0862		0.0000
Brazil	0.0311	0.0626	0.0558	0.0483	0.0237	0.0052	0.1057	0.0883	0.0836	0.0020	-0.0006	0.0706	

Below diagonal: Pairwise Fst values

Above diagonal: P-values P<0.0006 (after correction: 0.05/78) shaded

Supplementary Table S14. Pairwise Fst values calculated for DXS7424 and DXS101

DXS7424	Sinhala	SL Tamil	IND Tamil	Moors	Pakistan	China	Japan	Taiwan	Germany	Italy	Ivory Coast	Brazil
Sinhala		0.0533	0.0010	0.5079	0.0000	0.0000	0.0000	0.0000	0.0000	0.0000	0.0003	0.0000
SL Tamil	0.0035		0.2327	0.3984	0.0000	0.0000	0.0000	0.0000	0.0000	0.0000	0.0031	0.0000
IND Tamil	0.0112	0.0016		0.0509	0.0000	0.0000	0.0000	0.0000	0.0000	0.0000	0.0003	0.0000
Moors	-0.0005	0.0001	0.0066		0.0023	0.0000	0.0000	0.0000	0.0000	0.0000	0.0002	0.0000
Pakistan	0.0138	0.0285	0.0257	0.0117		0.0000	0.0000	0.0000	0.0005	0.0011	0.0000	0.0049
China	0.0783	0.1077	0.1019	0.0780	0.0286		0.0003	0.8338	0.0000	0.0714	0.0000	0.0011
Japan	0.0835	0.1154	0.1157	0.0871	0.0428	0.0082		0.0074	0.0000	0.0412	0.0000	0.0000
Taiwan	0.0770	0.1071	0.1016	0.0784	0.0292	-0.0014	0.0074		0.0000	0.1022	0.0000	0.0043
Germany	0.0355	0.0466	0.0399	0.0269	0.0082	0.0227	0.0311	0.0247		0.0015	0.0000	0.0722
Italy	0.0495	0.0735	0.0713	0.0479	0.0142	0.0035	0.0049	0.0036	0.0084		0.0000	0.2985
Ivory Coast	0.0135	0.0137	0.0229	0.0203	0.0506	0.1387	0.1340	0.1385	0.0727	0.0952		0.0000
Brazil	0.0425	0.0593	0.0530	0.0366	0.0083	0.0086	0.0180	0.0095	0.0017	0.0005	0.0887	

Below diagonal: Pairwise Fst values; Above diagonal: P-values with shaded cells indicating P<0.0008 (after correction:0.05/66)

DXS101	Sinhala	SL Tamil	IND Tamil	Moors	Brahmin India	Pakistan	China	Japan	Taiwan	Germany	Italy	Ivory Coast	Brazil
Sinhala		0.0664	0.1833	0.1847	0.2834	0.2602	0.0231	0.0355	0.0241	0.0000	0.0000	0.0000	0.0000
SL Tamil	0.0031		0.7313	0.2022	0.3611	0.0946	0.0730	0.0531	0.0154	0.0000	0.0036	0.0000	0.0011
IND Tamil	0.0015	-0.0018		0.2122	0.6142	0.2745	0.2996	0.5425	0.4450	0.0003	0.0016	0.0000	0.0001
Moors	0.0016	0.0019	0.0019		0.0416	0.0661	0.0468	0.0318	0.0062	0.0000	0.0001	0.0000	0.0000
Brahmin India	0.0014	0.0005	-0.0024	0.0110		0.6209	0.4240	0.4995	0.5332	0.0128	0.0351	0.0000	0.0156
Pakistan	0.0006	0.0029	0.0008	0.0040	-0.0021		0.2366	0.0486	0.0855	0.0005	0.0040	0.0000	0.0004
China	0.0023	0.0026	0.0006	0.0038	-0.0002	0.0006		0.2658	0.1266	0.0000	0.0000	0.0000	0.0000
Japan	0.0023	0.0035	-0.0006	0.0048	-0.0011	0.0028	0.0003		0.3863	0.0000	0.0000	0.0000	0.0000
Taiwan	0.0038	0.0067	-0.0002	0.0092	-0.0013	0.0027	0.0014	0.0000		0.0000	0.0000	0.0000	0.0000
Germany	0.0119	0.0094	0.0087	0.0126	0.0097	0.0051	0.0138	0.0176	0.0158		0.3334	0.0000	0.0006
Italy	0.0158	0.0089	0.0114	0.0165	0.0092	0.0076	0.0178	0.0227	0.0223	0.0002		0.0000	0.2800
Ivory Coast	0.0660	0.0491	0.0512	0.0597	0.0520	0.0450	0.0565	0.0684	0.0602	0.0319	0.0269		0.0000
Brazil	0.0193	0.0094	0.0125	0.0171	0.0109	0.0099	0.0171	0.0223	0.0247	0.0047	0.0006	0.0292	

Below diagonal: Pairwise Fst values; Above diagonal: P-values with shaded cells indicating P<0.0006 (after correction:0.05/78)

Supplementary Table S15. Haplotype frequencies observed among the Sinhalese male population for the four clusters of linked loci

Cluster I				Cluster II					Cluster III				Cluster IV			
DXS 10148	DXS 10135	DXS 8378	Freq.	DXS 7132	DXS 10079	DXS 10074	DXS 10075	Freq.	DXS 6801	DXS 6809	DXS 6789	Freq.	DXS 7424	DXS 101	DXS 7133	Freq.
17	20	11	0.0039	11	17	18	16	0.0039	8	32	22	0.0039	10	24	9	0.0078
17	23	11	0.0039	11	19	16	15	0.0039	9	34	16	0.0039	11	23	9	0.0039
17	25	10	0.0039	11	19	16	18	0.0039	10	31	15	0.0039	11	24	9	0.0155
17	27	12	0.0039	11	20	17	18	0.0039	10	31	20	0.0039	11	24	10	0.0116
17	29	11	0.0078	11	21	16	16	0.0039	10	31	21	0.0039	11	24	11	0.0116
17	31	11	0.0039	12	15	16	16	0.0039	10	32	15	0.0078	11	26	9	0.0078
18	16	12	0.0039	12	16	16	16	0.0039	10	32	16	0.0039	11	26	11	0.0039
18	18	11	0.0039	12	16	17	17	0.0039	10	32	20	0.0194	11	27	8	0.0039
18	18	12	0.0078	12	17	15	17	0.0039	10	32	22	0.0078	11	27	10	0.0039
18	19	10	0.0039	12	17	16	18	0.0039	10	32	23	0.0039	11	27	11	0.0078
18	20	11	0.0078	12	18	7	13	0.0116	10	33	14	0.0116	11	28	11	0.0039
18	21	9	0.0039	12	18	17	17	0.0116	10	33	15	0.0078	12	22	9	0.0116
18	21	10	0.0078	12	19	7	13	0.0078	10	33	16	0.0078	12	22	11	0.0039
18	22	9	0.0194	12	19	15	18	0.0078	10	33	20	0.0388	12	23	9	0.0039
18	22	12	0.0039	12	19	15	17	0.0039	10	33	21	0.0078	12	25	9	0.0078
18	23	11	0.0039	12	19	16	15	0.0039	10	33	22	0.0116	12	25	10	0.0078
18	24	10	0.0078	12	19	16	18	0.0039	10	33	23	0.0039	12	26	9	0.0039
18	24	11	0.0039	12	19	16	16	0.0039	10	34	15	0.0116	12	26	10	0.0039
18	25	10	0.0078	12	19	17	18	0.0039	10	34	16	0.0039	12	27	10	0.0039
18	25	11	0.0039	12	19	18	18	0.0039	10	34	20	0.0271	12	28	9	0.0116
18	25	12	0.0039	12	19	18	16	0.0039	10	34	21	0.0155	13	18	9	0.0039
18	26	9	0.0116	12	19	19	17	0.0039	10	35	15	0.0039	13	20	8	0.0039
18	26	10	0.0039	12	20	15	16	0.0039	10	35	16	0.0039	13	20	9	0.0039
18	26	11	0.0078	12	20	15	17	0.0039	10	35	20	0.0039	13	22	11	0.0078
18	26	12	0.0039	12	20	15	18	0.0039	10	35	21	0.0039	13	23	9	0.0116
18	27	10	0.0039	12	20	16	17	0.0039	10	35	22	0.0039	13	23	10	0.0039
18	27	11	0.0155	12	20	17	17	0.0039	10	36	15	0.0039	13	24	8	0.0039
18	28	10	0.0078	12	20	17	15	0.0039	10	36	20	0.0078	13	24	9	0.0504
18	28	11	0.0039	12	20	18	18	0.0116	10	36	21	0.0039	13	24	11	0.0349
18	28	12	0.0039	12	21	15	18	0.0039	10	36	23	0.0039	13	24	14	0.0078
18	29	10	0.0039	12	21	16	17	0.0039	11	29	15	0.0116	13	25	8	0.0078
18	29	11	0.0039	12	21	19	18	0.0039	11	29	19	0.0039	13	25	9	0.0349
18	30	11	0.0039	12	22	16	16	0.0039	11	29	20	0.0078	13	25	10	0.0155

18	31	12	0.0039	13	15	14	18	0.0039	11	30	15	0.0116	13	25	11	0.0039
18	32	12	0.0039	13	16	16	16	0.0039	11	30	20	0.0116	13	25	14	0.0078
18	33	9	0.0039	13	16	16	18	0.0078	11	30	23	0.0078	13	26	9	0.0155
18	33	11	0.0039	13	17	17	17	0.0039	11	31	15	0.0078	13	26	11	0.0116
18	33	12	0.0039	13	17	20	16	0.0039	11	31	16	0.0078	13	27	8	0.0039
18	34	12	0.0039	13	18	15	18	0.0039	11	31	20	0.0039	13	27	9	0.0078
18	37	11	0.0039	13	18	16	17	0.0155	11	31	21	0.0039	13	27	10	0.0039
19	18	12	0.0039	13	18	16	18	0.0078	11	31	23	0.0039	13	28	11	0.0039
19	19	11	0.0039	13	18	17	17	0.0078	11	32	15	0.0039	13	28	13	0.0039
19	22	9	0.0078	13	18	17	18	0.0039	11	32	16	0.0039	13	30	10	0.0039
19	22	10	0.0039	13	18	17.1	17	0.0039	11	32	20	0.0271	14	22	9	0.0039
19	22	12	0.0039	13	18	18	18	0.0039	11	32	21	0.0078	14	22	10	0.0039
19	23	9	0.0039	13	18	18	17	0.0039	11	32	22	0.0116	14	23	9	0.0039
19	23	11	0.0039	13	19	8	16	0.0039	11	33	14	0.0039	14	24	9	0.0194
19	24	12	0.0039	13	19	9	17	0.0039	11	33	15	0.0349	14	24	10	0.0078
19	25	12	0.0039	13	19	15	19	0.0039	11	33	16	0.0078	14	24	11	0.0155
19	27	11	0.0039	13	19	15	17	0.0078	11	33	18	0.0078	14	25	8	0.0039
19	28	11	0.0039	13	19	16	19	0.0039	11	33	20	0.0388	14	25	9	0.0233
19	29	11	0.0039	13	19	16	18	0.0039	11	33	21	0.0233	14	25	10	0.0116
19	31	11	0.0039	13	19	16	17	0.0039	11	33	22	0.0078	14	25	11	0.0155
20	18	11	0.0116	13	19	17	17	0.0078	11	33	23	0.0039	14	25	15	0.0039
20	20	12	0.0078	13	19	17	18	0.0078	11	34	14	0.0039	14	26	9	0.0155
20	21	12	0.0078	13	19	18	18	0.0039	11	34	15	0.0271	14	26	10	0.0039
20	23	11	0.0039	13	19	18	16	0.0039	11	34	16	0.0194	14	26	11	0.0039
20	24	12	0.0039	13	19	18	17	0.0039	11	34	17	0.0039	14	27	9	0.0116
20	27	12	0.0039	13	19	19	17	0.0078	11	34	20	0.0155	14	27	11	0.0078
20	28	10	0.0039	13	20	8	18	0.0039	11	34	21	0.0194	14	28	9	0.0078
20	31	11	0.0039	13	20	16	18	0.0078	11	34	22	0.0116	14	28	10	0.0039
20	34	10	0.0039	13	20	16	17	0.0078	11	34	23	0.0039	14	28	11	0.0078
20.1	21	12	0.0039	13	20	17	18	0.0039	11	34	24	0.0039	14	29	9	0.0039
20.1	26	11	0.0039	13	20	17	16	0.0078	11	35	15	0.0039	14	31	11	0.0039
20.1	29	11	0.0039	13	20	17	17	0.0078	11	35	16	0.0039	15	18	11	0.0039
21	14	12	0.0039	13	20	18	17	0.0194	11	35	21	0.0116	15	20	9	0.0039
21	17	11	0.0039	13	20	18	18	0.0078	11	35	22	0.0155	15	22	9	0.0039
21	21	11	0.0078	13	20	18	19	0.0039	11	35	23	0.0039	15	23	9	0.0078
21	23	11	0.0039	13	20	18	16	0.0078	11	36	16	0.0078	15	23	10	0.0078
21	24	11	0.0078	13	20	19	16	0.0039	11	36	20	0.0078	15	23	12	0.0039

21	24	12	0.0039	13	21	16	16	0.0039	11	36	21	0.0039	15	24	9	0.0271
21	26	11	0.0039	13	21	17	18	0.0039	11	36	22	0.0039	15	24	10	0.0039
21	28	10	0.0039	13	21	17	15	0.0039	12	31	15	0.0155	15	24	11	0.0116
21.1	29	12	0.0078	13	21	17	17	0.0078	12	31	17	0.0039	15	24	12	0.0078
21.1	30	11	0.0039	13	21	18	18	0.0039	12	31	20	0.0194	15	24	14	0.0039
22	26	10	0.0039	13	21	18	16	0.0039	12	31	21	0.0039	15	25	9	0.0233
22.1	21	10	0.0039	13	21	19	16	0.0039	12	31	22	0.0039	15	25	10	0.0116
22.1	24	11	0.0039	13	21	20	18	0.0039	12	32	18	0.0039	15	25	11	0.0078
22.1	25	11	0.0039	13	22	16	17	0.0039	12	32	20	0.0194	15	26	9	0.0116
22.1	28	10	0.0039	13	22	17	17	0.0039	12	32	22	0.0039	15	26	10	0.0078
22.1	28	11	0.0039	13	22	17	18	0.0039	12	33	14	0.0039	15	26	11	0.0078
22.1	28	12	0.0039	13	22	19	17	0.0039	12	33	15	0.0194	15	26	12	0.0078
22.1	29	10	0.0039	14	13	17	18	0.0039	12	33	16	0.0155	15	27	9	0.0233
22.1	30	11	0.0039	14	15	17	16	0.0039	12	33	17	0.0078	15	27	11	0.0039
23.1	21	10	0.0039	14	15	17	18	0.0078	12	33	20	0.0349	15	28	9	0.0039
23.1	21	11	0.0039	14	15	18	17	0.0039	12	33	21	0.0078	15	28	10	0.0039
23.1	24	10	0.0039	14	15	19	18	0.0039	12	33	22	0.0116	15	28	11	0.0039
23.1	24	13	0.0039	14	16	15	16	0.0039	12	34	15	0.0116	15	28	13	0.0039
23.1	26	11	0.0078	14	16	16	16	0.0078	12	34	20	0.0233	15	30	11	0.0039
23.1	27	11	0.0078	14	16	17	17	0.0039	12	34	22	0.0039	16	18	11	0.0039
23.1	29	12	0.0039	14	16	18	17	0.0039	12	35	15	0.0078	16	22	10	0.0039
23.1	30	12	0.0039	14	17	16	16	0.0039	12	35	20	0.0039	16	22	11	0.0039
23.1	38	12	0.0039	14	17	16	17	0.0039	12	35	21	0.0039	16	24	9	0.0310
23.2	25	10	0.0039	14	17	17	18	0.0039	12	36	14	0.0039	16	24	10	0.0116
24.1	19	11	0.0039	14	17	18	17	0.0039	12	36	22	0.0039	16	24	11	0.0039
24.1	19	12	0.0039	14	17	18	16	0.0039	12	37	15	0.0039	16	25	8	0.0039
24.1	20	10	0.0039	14	18	15	16	0.0039	12	37	20	0.0039	16	25	9	0.0233
24.1	21	10	0.0039	14	18	16	16	0.0078	12	37	21	0.0039	16	25	14	0.0078
24.1	22	9	0.0078	14	18	17	16	0.0078	12	38	14	0.0039	16	26	9	0.0194
24.1	22	10	0.0039	14	18	17	17	0.0078	13	31	16	0.0039	16	26	11	0.0078
24.1	22	11	0.0078	14	18	18	18	0.0039	13	32	15	0.0039	16	27	9	0.0194
24.1	23	9	0.0039	14	18	19	19	0.0039	13	32	19	0.0039	16	27	11	0.0039
24.1	23	10	0.0078	14	19	8	17	0.0039	13	33	15	0.0039	16	28	9	0.0039
24.1	24	9	0.0039	14	19	9	16	0.0039	13	33	20	0.0116	16	28	11	0.0155
24.1	24	10	0.0039	14	19	15	17	0.0039	13	33	21	0.0039	16	29	9	0.0039
24.1	24	12	0.0039	14	19	16	19	0.0039	13	33	22	0.0039	17	15	9	0.0039
24.1	25	10	0.0039	14	19	16	16	0.0039	13	33	23	0.0039	17	21	11	0.0078

24.1	25	11	0.0078
24.1	25	12	0.0039
24.1	26	11	0.0039
24.1	26	12	0.0039
24.1	27	11	0.0078
24.1	28	10	0.0039
24.1	28	11	0.0039
24.1	29	10	0.0039
24.1	33	10	0.0039
24.1	34	10	0.0039
25.1	17	12	0.0039
25.1	19	11	0.0039
25.1	20	10	0.0039
25.1	20	12	0.0078
25.1	21	10	0.0078
25.1	23	9	0.0039
25.1	24	11	0.0039
25.1	25	10	0.0078
25.1	25	12	0.0039
25.1	26	10	0.0039
25.1	26	11	0.0078
25.1	27	10	0.0039
25.1	27	11	0.0078
25.1	27	12	0.0039
25.1	28	11	0.0078
25.1	29	11	0.0078
25.1	29	12	0.0039
25.1	30	11	0.0039
25.1	30	12	0.0039
25.1	33	11	0.0039
26	27	12	0.0039
26.1	20	10	0.0039
26.1	20	11	0.0039
26.1	21	10	0.0039
26.1	21	11	0.0039
26.1	22	10	0.0155
26.1	22	11	0.0039

14	19	17	18	0.0039
14	19	17	17	0.0155
14	19	17	16	0.0039
14	19	18	17	0.0233
14	19	18	16	0.0116
14	19	18	18	0.0039
14	19	19	18	0.0078
14	19	19	17	0.0078
14	20	7	13	0.0039
14	20	15	17	0.0039
14	20	15.3	18	0.0039
14	20	16	16	0.0194
14	20	16	17	0.0078
14	20	16	15	0.0039
14	20	16	18	0.0078
14	20	17	17	0.0155
14	20	17.3	17	0.0039
14	20	18	16	0.0116
14	20	18	17	0.0116
14	20	18	18	0.0039
14	20	19	16	0.0039
14	20	19	19	0.0078
14	20	19	18	0.0039
14	20	20	17	0.0039
14	21	15	18	0.0039
14	21	16	15	0.0039
14	21	16	16	0.0194
14	21	16	17	0.0116
14	21	17	16	0.0078
14	21	17	17	0.0116
14	21	18	18	0.0039
14	21	18	16	0.0078
14	21	19	17	0.0039
14	22	16	16	0.0039
14	22	18	16	0.0078
14	22	18	17	0.0078
14	22	18	18	0.0039

13	34	18	0.0039
13	34	21	0.0078
13	35	22	0.0039
13	36	15	0.0039
13	36	21	0.0039
13	36	22	0.0039

17	22	11	0.0039
17	24	9	0.0039
17	24	10	0.0039
17	24	11	0.0078
17	26	11	0.0039
18	15	10	0.0039
18	25	9	0.0039
19	25	9	0.0039

26.1	23	13	0.0039
26.1	24	11	0.0039
26.1	25	11	0.0116
26.1	26	10	0.0078
26.1	26	12	0.0039
26.1	27	11	0.0155
26.1	27	12	0.0039
26.1	27	13	0.0039
26.1	28	11	0.0116
26.1	29	9	0.0039
26.1	29	10	0.0039
26.1	29	11	0.0039
26.1	29	13	0.0039
26.1	30	12	0.0078
26.1	32	11	0.0039
27.1	17	12	0.0078
27.1	18	13	0.0039
27.1	20	9	0.0039
27.1	20	10	0.0039
27.1	21	11	0.0078
27.1	21	14	0.0039
27.1	22	10	0.0039
27.1	22	12	0.0039
27.1	23	11	0.0039
27.1	24	9	0.0039
27.1	24	10	0.0039
27.1	24	13	0.0078
27.1	25	10	0.0039
27.1	26	11	0.0078
27.1	26	12	0.0039
27.1	27	11	0.0116
27.1	28	10	0.0039
27.1	28	11	0.0078
27.1	28	12	0.0039
27.1	29	12	0.0039
27.1	30	11	0.0039
27.1	32	11	0.0039

14	22	19	16	0.0039
14	22	19	17	0.0078
14	22	21	17	0.0039
15	16	15	16	0.0039
15	17	15	17	0.0039
15	17	16	17	0.0039
15	18	16	16	0.0078
15	18	16	17	0.0078
15	18	17	18	0.0039
15	18	17	17	0.0039
15	18	18	17	0.0039
15	19	15	16	0.0039
15	19	16	18	0.0039
15	19	19	18	0.0039
15	20	17	16	0.0039
15	20	17	17	0.0039
15	20	18	18	0.0039
15	20	18	17	0.0039
15	20	19	17	0.0155
15	20	19	18	0.0039
15	20	20	17	0.0039
15	21	15	17	0.0039
15	21	16	17	0.0039
15	21	17	18	0.0039
15	21	17	16	0.0039
15	21	18	17	0.0078
15	21	19	18	0.0039
15	22	19	17	0.0039
16	17	16	16	0.0039
16	17	16	17	0.0039
16	18	16	16	0.0039
16	18	16	19	0.0039
16	19	15	17	0.0039
16	20	16	20	0.0039
16	22	18	17	0.0039

27.1	37	11	0.0039
28.1	20	11	0.0039
28.1	21	11	0.0039
28.1	22	10	0.0039
28.1	22	11	0.0039
28.1	23	10	0.0078
28.1	23	11	0.0039
28.1	23	12	0.0039
28.1	27	11	0.0116
28.1	29	10	0.0039
28.1	31	11	0.0039
28.1	35	11	0.0039
29.1	20	12	0.0039
29.1	22	9	0.0039
29.1	25	13	0.0039
31.1	29	12	0.0039

Supplementary Table S16. Haplotype frequencies observed among the Sri Lankan Tamil male population for the four clusters of linked loci

Cluster I				Cluster II					Cluster III				Cluster IV			
DXS 10148	DXS 10135	DXS 8378	Freq.	DXS 7132	DXS 10079	DXS 10074	DXS 10075	Freq.	DXS 6801	DXS 6809	DXS 6789	Freq.	DXS 7424	DXS 101	DXS 7133	Freq.
17	22	10	0.0130	12	17	8	17	0.0130	8	34	16	0.0130	11	22	9	0.0130
17	22	12	0.0130	12	18	7	13	0.0130	10	30	16	0.0130	11	23	9	0.0130
17	29	11	0.0130	12	18	18	18	0.0130	10	32	15	0.0130	11	23	10	0.0260
18	22	12	0.0260	12	19	7	13	0.0130	10	32	16	0.0130	11	24	9	0.0130
18	25	10	0.0130	12	19	20	17	0.0130	10	33	15	0.0519	11	24	11	0.0130
18	25	11	0.0130	12	20	15	16	0.0130	10	33	20	0.0260	11	25	9	0.0130
18	27	11	0.0260	12	20	17	17	0.0130	10	33	21	0.0260	11	25	11	0.0130
18	28	10	0.0130	13	15	16	16	0.0130	10	34	14	0.0130	11	26	9	0.0130
18	30	12	0.0130	13	16	15	17	0.0130	10	34	15	0.0260	11	26	10	0.0260
18	30	13	0.0130	13	17	16	17	0.0130	10	34	16	0.0130	11	27	9	0.0130
18	31	11	0.0130	13	17	17	16	0.0130	10	35	20	0.0130	12	26	9	0.0130
18	32	12	0.0130	13	18	18	17	0.0130	10	36	16	0.0130	13	18	11	0.0130
18	32	14	0.0130	13	19	16	18	0.0260	10	36	20	0.0130	13	21	8	0.0130
18	37	12	0.0130	13	19	17	16	0.0130	10	37	20	0.0130	13	23	8	0.0130
19	19	10	0.0130	13	19	18	17	0.0260	11	29	21	0.0130	13	23	10	0.0130
19	21.1	11	0.0130	13	19	19	18	0.0130	11	31	15	0.0130	13	24	9	0.0390
19	23	9	0.0130	13	20	16	17	0.0130	11	31	21	0.0130	13	24	10	0.0130
20	22	11	0.0130	13	21	7	14	0.0130	11	31	22	0.0130	13	24	11	0.0260
20	25	10	0.0130	13	21	17	16	0.0130	11	32	15	0.0130	13	24	12	0.0130
20	26	11	0.0130	13	21	18	18	0.0130	11	32	16	0.0130	13	25	9	0.0390
20	30	11	0.0130	14	18	15	17	0.0130	11	32	20	0.0260	13	25	10	0.0130
21	31	11	0.0130	14	18	16	17	0.0130	11	32	21	0.0130	13	25	11	0.0130
21.1	21	10	0.0130	14	18	18	16	0.0130	11	33	14	0.0130	13	25	14	0.0130
22	28	11	0.0130	14	18	18	17	0.0130	11	33	15	0.0260	13	26	10	0.0130
23.1	17	11	0.0130	14	18	19	17	0.0130	11	33	16	0.0260	13	27	9	0.0130
23.1	18	11	0.0130	14	19	16	17	0.0260	11	33	20	0.0130	13	27	10	0.0130
23.1	22	9	0.0130	14	19	17	17	0.0390	11	33	22	0.0130	13	28	9	0.0130
23.1	25	10	0.0130	14	19	17	18	0.0130	11	34	15	0.0260	14	21	8	0.0130
23.1	26	12	0.0130	14	19	18	19	0.0130	11	34	16	0.0130	14	23	10	0.0130
23.1	29	10	0.0130	14	20	15	18	0.0130	11	34	20	0.0130	14	23	11	0.0130
24.1	17	11	0.0130	14	20	16	17	0.0130	11	34	21	0.0130	14	24	9	0.0390
24.1	18	12	0.0130	14	20	17	17	0.0390	11	34	22	0.0130	14	24	10	0.0130
24.1	22	12	0.0130	14	20	17	18	0.0130	11	34	23	0.0130	14	25	9	0.0649
24.1	25	12	0.0130	14	20	18	16	0.0260	11	35	14	0.0130	14	25	10	0.0130
24.1	38	14	0.0130	14	20	19	16	0.0130	11	35	21	0.0130	14	25	13	0.0130
25.1	16	11	0.0130	14	21	15	16	0.0130	11	35	23	0.0130	14	26	9	0.0130

25.1	19	11	0.0130	14	21	17	16	0.0260	11	36	15	0.0130	14	26	10	0.0130
25.1	20	10	0.0130	14	21	18	17	0.0519	11	36	21	0.0130	14	27	9	0.0260
25.1	20	12	0.0260	14	21	18	18	0.0130	12	28	20	0.0130	14	28	9	0.0130
25.1	22	11	0.0130	14	21	19	16	0.0130	12	31	19	0.0130	15	15	14	0.0130
25.1	24	11	0.0390	14	22	16	18	0.0130	12	31	20	0.0130	15	19	9	0.0130
25.1	25	11	0.0130	14	22	18	17	0.0130	12	32	15	0.0519	15	23	9	0.0260
25.1	25	14	0.0130	14	22	19	16	0.0130	12	33	15	0.0260	15	23	10	0.0130
25.1	31	12	0.0130	14	23	16	17	0.0130	12	33	16	0.0130	15	23	11	0.0130
25.1	32	11	0.0130	15	15	16	16	0.0130	12	33	17	0.0130	15	24	9	0.0260
26	21	11	0.0130	15	16	15	17	0.0130	12	34	15	0.0390	15	24	11	0.0130
26.1	17	12	0.0130	15	17	16	17	0.0130	12	34	20	0.0130	15	25	9	0.0260
26.1	18	10	0.0130	15	17	17	16	0.0130	12	34	21	0.0260	15	25	11	0.0130
26.1	18	12	0.0130	15	17	18	16	0.0130	12	35	16	0.0130	15	26	9	0.0519
26.1	19	11	0.0130	15	18	17	17	0.0130	12	35	20	0.0260	15	26	11	0.0130
26.1	20	9	0.0130	15	19	16	16	0.0130	13	31	16	0.0130	15	27	9	0.0260
26.1	20	10	0.0130	15	19	16	17	0.0260	13	32	15	0.0260	15	28	10	0.0130
26.1	23	10	0.0130	15	19	16	18	0.0130	13	33	15	0.0260	16	15	10	0.0130
26.1	24	11	0.0130	15	19	17	18	0.0130	13	33	16	0.0130	16	22	11	0.0130
26.1	25	11	0.0130	15	20	15	17	0.0130	13	34	20	0.0130	16	25	9	0.0130
26.1	27	11	0.0260	15	20	16	17	0.0260	13	35	20	0.0130	16	28	11	0.0130
26.1	27	12	0.0130	15	20	17	17	0.0130	13	35	22	0.0130				
26.1	29	11	0.0130	15	20	18	17	0.0130								
26.1	30	12	0.0130	15	21	18	16	0.0130								
27.1	17	12	0.0130	16	19	18	17	0.0130								
27.1	21	12	0.0130	16	19	19	17	0.0130								
27.1	22.1	10	0.0130	16	20	15	17	0.0130								
27.1	28	11	0.0130	16	21	18	17	0.0130								
27.1	29	11	0.0130													
27.1	32	10	0.0130													
27.1	32	11	0.0130													
28.1	24	10	0.0130													
28.1	24	11	0.0260													
30.1	19	12	0.0130													
30.1	24	12	0.0130													

Supplementary Table S17. Haplotype frequencies observed among the Indian Tamil male population for the four clusters of linked loci

Cluster I				Cluster II					Cluster III				Cluster IV			
DXS 10148	DXS 10135	DXS 8378	Freq.	DXS 7132	DXS 10079	DXS 10074	DXS 10075	Freq.	DXS 6801	DXS 6809	DXS 6789	Freq.	DXS 7424	DXS 101	DXS 7133	Freq.
17	20	10	0.0159	11	19	7	13	0.0159	9	33	20	0.0159	11	23	10	0.0159
17	30	13	0.0159	11	20	17	17	0.0159	10	30	20	0.0159	11	24	10	0.0159
17	34	12	0.0159	12	18	7	13	0.0317	10	31	15	0.0317	12	24	9	0.0159
18	20	9	0.0159	12	18	18	17	0.0159	10	31	16	0.0159	12	25	9	0.0159
18	26	11	0.0159	12	19	17	16	0.0159	10	31	18	0.0159	13	23	11	0.0159
18	27	11	0.0317	12	20	17	17	0.0159	10	31	23	0.0159	13	24	8	0.0159
18	29	11	0.0159	12	20	19	17	0.0159	10	32	16	0.0159	13	24	9	0.0317
18	31	11	0.0159	12	22	19	17	0.0159	10	32	20	0.0317	13	24	10	0.0159
18	32	11	0.0159	12	23	18	19	0.0159	10	33	21	0.0317	13	24	11	0.0476
18	38	13	0.0159	13	17	18	16	0.0159	10	33	22	0.0159	13	25	10	0.0159
19	24	11	0.0159	13	18	7	13	0.0159	10	34	20	0.0317	13	25	11	0.0159
19	28	10	0.0159	13	18	15	18	0.0159	10	35	15	0.0476	13	26	9	0.0159
19	33	11	0.0159	13	18	17	17	0.0159	10	35	20	0.0159	13	26	10	0.0317
20	19	11	0.0159	13	18	17	18	0.0159	10	35	21	0.0159	13	28	9	0.0159
20	20	9	0.0159	13	18	18	17	0.0159	11	28	15	0.0159	13	28	10	0.0159
20	20	10	0.0159	13	19	14	17	0.0159	11	30	15	0.0317	14	21	9	0.0159
20	26	11	0.0317	13	19	16	17	0.0317	11	31	15	0.0317	14	22	9	0.0159
20	27	10	0.0159	13	19	17	17	0.0159	11	31	20	0.0159	14	23	9	0.0159
21	19	10	0.0159	13	20	15	17	0.0159	11	32	21	0.0159	14	23	11	0.0159
21	34	12	0.0159	13	20	17	18	0.0159	11	32	22	0.0159	14	24	9	0.0159
21.1	22	11	0.0159	13	20	18	18	0.0159	11	33	14	0.0159	14	24	11	0.0317
22	25	10	0.0159	13	22	12	16	0.0159	11	33	15	0.0159	14	25	9	0.0159
22.1	30	12	0.0159	13	22	15	17	0.0159	11	33	16	0.0476	14	25	12	0.0159
23.1	18	11	0.0159	14	16	18	16	0.0159	11	33	18	0.0159	14	26	9	0.0159
23.1	25	10	0.0159	14	18	18	18	0.0159	11	33	20	0.0476	14	26	10	0.0159
23.1	33	11	0.0159	14	18	19	18	0.0159	11	33	21	0.0317	14	26	11	0.0317
24.1	21	11	0.0317	14	19	16	17	0.0476	11	34	15	0.0317	14	27	9	0.0159
24.1	23	11	0.0159	14	19	16	18	0.0159	11	34	21	0.0159	15	23	9	0.0476
24.1	24	12	0.0159	14	19	17	18	0.0159	11	34	22	0.0159	15	24	9	0.0317
24.1	26	10	0.0159	14	19	18	16	0.0159	11	35	20	0.0159	15	24	11	0.0317
24.1	26	11	0.0159	14	19	18	17	0.0159	11	36	20	0.0159	15	25	9	0.0159

25.1	18	9	0.0159	14	19	19	17	0.0159	11	36	21	0.0159	15	25	10	0.0317
25.1	22	9	0.0159	14	20	16	17	0.0476	12	31	15	0.0159	15	25	11	0.0159
25.1	24	9	0.0159	14	20	17	16	0.0159	12	32	15	0.0159	15	25	12	0.0159
25.1	24	11	0.0317	14	20	17	17	0.0317	12	32	20	0.0159	15	26	8	0.0159
25.1	26	10	0.0159	14	20	18	17	0.0159	12	33	15	0.0159	15	26	9	0.0159
25.1	27	11	0.0159	14	20	19	17	0.0159	12	33	19	0.0159	15	26	11	0.0317
25.1	33	12	0.0159	14	21	15	18	0.0159	12	33	21	0.0159	15	27	9	0.0317
26.1	18	13	0.0159	14	21	18	17	0.0159	12	34	15	0.0159	15	28	9	0.0159
26.1	21	11	0.0159	14	21	19	17	0.0159	12	34	16	0.0159	16	18	9	0.0159
26.1	23	12	0.0159	15	15	18	18	0.0159	12	35	20	0.0635	16	23	9	0.0159
26.1	24	9	0.0159	15	17	18	16	0.0159	12	36	15	0.0159	16	24	9	0.0159
26.1	25	12	0.0317	15	18	7	13	0.0159	12	36	20	0.0159	16	24	12	0.0159
26.1	27	12	0.0159	15	18	17	17	0.0159	13	32	20	0.0159	16	27	10	0.0159
26.1	28	11	0.0159	15	19	8	15	0.0159	13	33	16	0.0159	16	28	10	0.0159
26.1	29	12	0.0159	15	19	16	16	0.0159	13	33	21	0.0159	16	28	11	0.0159
26.1	31	10	0.0159	15	19	16	17	0.0159					17	18	9	0.0159
27.1	19	10	0.0159	15	19	20	17	0.0159					17	24	10	0.0159
27.1	20	11	0.0159	15	20	8	15	0.0159					18	19	9	0.0159
27.1	21	10	0.0159	15	20	16	18	0.0159					18	27	9	0.0159
27.1	26	11	0.0317	15	20	18	17	0.0159								
27.1	28	9	0.0159	15	21	15	16	0.0159								
27.1	30	11	0.0317	15	21	18	17	0.0159								
28.1	24	10	0.0159	16	19	17	16	0.0159								
28.1	30	11	0.0159	16	19	17	17	0.0159								
29.1	32	11	0.0159	17	20	18	18	0.0159								

Supplementary Table S18. Haplotype frequencies observed among the Moor male population for the four clusters of linked loci

Cluster I				Cluster II					Cluster III				Cluster IV			
DXS 10148	DXS 10135	DXS 8378	Freq.	DXS 7132	DXS 10079	DXS 10074	DXS 10075	Freq.	DXS 6801	DXS 6809	DXS 6789	Freq.	DXS 7424	DXS 101	DXS 7133	Freq.
18	22	10	0.0149	12	18	7	14	0.0149	9	31	22	0.0149	11	23	9	0.0149
18	22	11	0.0149	12	18	15	17	0.0149	10	30	15	0.0149	11	24	9	0.0299
18	22	12	0.0149	12	18	16	17	0.0149	10	31	20	0.0149	11	24	11	0.0149
18	24	10	0.0149	12	18	17	18	0.0149	10	32	15	0.0149	11	25	10	0.0149
18	27	11	0.0149	12	19	15	17	0.0149	10	33	15	0.0299	12	24	9	0.0299
18	28	11	0.0149	12	19	19	17	0.0149	10	33	20	0.0299	12	25	9	0.0149
18	29	10	0.0149	12	20	7	13	0.0149	10	33	22	0.0299	12	26	10	0.0149
18	29	12	0.0149	12	20	17	16	0.0149	10	34	15	0.0597	12	26	11	0.0149
18	31	11	0.0149	12	20	18	16	0.0149	10	34	16	0.0149	12	28	9	0.0149
18	35	12	0.0149	12	20	18	17	0.0149	10	34	20	0.0299	12	29	10	0.0299
19	23	11	0.0149	12	21	16	17	0.0149	10	34	21	0.0149	13	23	8	0.0149
19	27	11	0.0149	13	16	18	17	0.0299	10	34	22	0.0149	13	23	10	0.0149
19.1	29	12	0.0149	13	17	18	16	0.0149	10	35	15	0.0149	13	25	9	0.0448
20	18	10	0.0149	13	18	9	14	0.0149	10	37	15	0.0149	13	25	11	0.0149
20	21	11	0.0149	13	18	16	18	0.0149	11	29	21	0.0149	13	27	9	0.0149
20	23	11	0.0149	13	18	19	17	0.0149	11	31	15	0.0299	13	29	11	0.0149
20	25	10	0.0299	13	19	16	15	0.0149	11	31	16	0.0149	14	22	9	0.0149
20	26	13	0.0149	13	19	16	18	0.0149	11	31	20	0.0149	14	24	9	0.0149
20	28	10	0.0149	13	19	17	17	0.0149	11	31	21	0.0149	14	24	10	0.0149
20	29	10	0.0149	13	19	19	18	0.0149	11	32	16	0.0149	14	24	11	0.0149
20	29	11	0.0149	13	20	8	17	0.0149	11	32	20	0.0448	14	25	10	0.0149
20.1	19	12	0.0149	13	20	17	17	0.0149	11	32	21	0.0149	14	25	11	0.0448
21	21	11	0.0149	13	20	18	16	0.0299	11	33	15	0.0448	14	26	9	0.0149
21	22	11	0.0149	13	20	18	17	0.0149	11	33	16	0.0597	14	26	11	0.0149
21	29	11	0.0149	13	20	18	19	0.0149	11	33	20	0.0448	14	27	9	0.0299
23.1	27	11	0.0149	13	21	8	19	0.0149	11	33	21	0.0149	14	27	13	0.0149
24.1	18	10	0.0149	13	21	16	18	0.0149	11	34	16	0.0149	14	28	9	0.0149
24.1	27	12	0.0149	13	21	18	17	0.0149	11	34	22	0.0149	15	23	9	0.0299
24.1	31	11	0.0149	13	22	17	17	0.0149	11	34	23	0.0149	15	23	11	0.0299
25.1	17	11	0.0149	14	16	17	16	0.0149	12	29	19	0.0149	15	24	9	0.0149
25.1	22	9	0.0149	14	18	9	14	0.0149	12	30	20	0.0299	15	24	11	0.0597
25.1	22	12	0.0149	14	18	15	17	0.0149	12	31	15	0.0149	15	25	9	0.0149
25.1	23	10	0.0149	14	18	17	12	0.0149	12	32	20	0.0149	15	25	11	0.0299

25.1	24	10	0.0149	14	18	18	12	0.0149	12	33	15	0.0149	15	27	9	0.0299
25.1	26	11	0.0149	14	18	19	17	0.0149	12	33	17	0.0149	15	27	11	0.0149
25.1	29	11	0.0149	14	19	15	17	0.0149	12	34	15	0.0149	15	28	11	0.0149
25.1	30	11	0.0149	14	19	16	18	0.0149	12	34	20	0.0299	16	18	10	0.0149
25.1	33	12	0.0149	14	19	17	18	0.0299	12	34	21	0.0149	16	19	9	0.0149
26.1	18	12	0.0149	14	20	16	16	0.0149	12	35	20	0.0149	16	23	10	0.0149
26.1	20	10	0.0149	14	20	16	18	0.0149	12	36	14	0.0149	16	23	11	0.0149
26.1	21	11	0.0149	14	20	17	18	0.0448	13	31	16	0.0149	16	24	9	0.0149
26.1	21	12	0.0149	14	21	15	17	0.0149	13	32	20	0.0149	16	25	9	0.0149
26.1	23	11	0.0149	14	21	18	16	0.0149	13	32	21	0.0149	16	25	10	0.0299
26.1	24	11	0.0149	14	21	19	16	0.0149	13	33	19	0.0149	16	26	9	0.0149
26.1	26	10	0.0149	14	22	16	17	0.0149	13	33	20	0.0149	16	28	9	0.0149
26.1	27	10	0.0149	14	22	17	18	0.0149	13	33	21	0.0149	16	29	11	0.0149
26.1	27	11	0.0299	14	22	19	18	0.0149	13	35	20	0.0149	17	24	9	0.0149
26.1	28	11	0.0149	15	17	17	18	0.0149	13	35	22	0.0149	17	24	10	0.0149
26.1	29	10	0.0149	15	18	16	18	0.0149					17	26	11	0.0149
26.1	29	12	0.0149	15	18	17	18	0.0149					17	26	13	0.0149
26.1	31	10	0.0448	15	18	18	17	0.0149					17	27	11	0.0149
26.1	31	11	0.0299	15	19	8	15	0.0149								
26.1	34	11	0.0149	15	19	16	16	0.0149								
27.1	21	13	0.0149	15	19	16	18	0.0149								
27.1	25	13	0.0149	15	19	17	15	0.0149								
28.1	21	12	0.0149	15	19	17	17	0.0149								
28.1	21.1	10	0.0149	15	19	19	17	0.0149								
28.1	22	12	0.0149	15	21	17	18	0.0149								
28.1	28	11	0.0149	15	21	20	17	0.0149								
28.1	28	12	0.0149	15	22	18	17	0.0149								
29.1	21	11	0.0149	16	18	17	16	0.0149								
29.1	30	11	0.0149	17	20	15	16	0.0149								

Supplementary Table S19. Forensic efficiency parameters for the four clusters

Cluster I DXS10148-DXS10135-DXS8378

Ethnicity	Sinhalese	SL Tamil	Indian Tamil	Moors
PIC	0.9936	0.9840	0.9802	0.9821
He:	0.9936	0.9843	0.9806	0.9824
PD female:	0.9999	0.9995	0.9993	0.9994
PD male:	0.9936	0.9843	0.9806	0.9824
MEC Krü:	0.9924	0.9694	0.9619	0.9633
MEC Des.trio:	0.9936	0.9840	0.9802	0.9821
MEC Des.duo:	0.9873	0.9689	0.9616	0.9652

Cluster II DXS7132-DXS10079-DXS10074-DXS10075

Ethnicity	Sinhalese	SL Tamil	Indian Tamil	Moors
PIC	0.9922	0.9802	0.9792	0.9821
He:	0.9923	0.9806	0.9796	0.9824
PD female:	0.9999	0.9993	0.9992	0.9994
PD male:	0.9936	0.9806	0.9796	0.9824
MEC Krü:	0.9891	0.9619	0.9601	0.9633
MEC Des.trio:	0.9922	0.9802	0.9792	0.9821
MEC Des.duo:	0.9846	0.9616	0.9596	0.9652

Cluster III DXS6801-DXS6809-DXS6789

Ethnicity	Sinhalese	SL Tamil	Indian Tamil	Moors
PIC	0.9838	0.9774	0.9718	0.9719
He:	0.9840	0.9779	0.9725	0.9726
PD female:	0.9995	0.9990	0.9985	0.9985
PD male:	0.9840	0.9779	0.9725	0.9726
MEC Krü:	0.9703	0.9563	0.9452	0.9444
MEC Des.trio:	0.9838	0.9774	0.9718	0.9719
MEC Des.duo:	0.9685	0.9564	0.9460	0.9462

Cluster IV DXS7424-DXS101-DXS7133

Ethnicity	Sinhalese	SL Tamil	Indian Tamil	Moors
PIC	0.9841	0.9753	0.9760	0.9752
He:	0.9843	0.9759	0.9766	0.9757
PD female:	0.9995	0.9989	0.9989	0.9989
PD male:	0.9843	0.9759	0.9766	0.9757
MEC Krü:	0.9711	0.9523	0.9534	0.9506
MEC Des.trio:	0.9841	0.9753	0.9760	0.9752
MEC Des.duo:	0.9690	0.9525	0.9538	0.9522

PIC: polymorphism information content, He: expected heterozygosity, PD female: power of discrimination in females, PD male: power of discrimination in males, MEC Krü: mean exclusion chance Kruger, MEC Des.trio: mean exclusion chance Desmaris trio. MEC Des.duo: mean exclusion chance Desmaris duo

Supplementary Table S20. Haplotype frequencies observed among the Sinhalese male population

DXS 7132	DXS 10079	DXS 10074	Freq.	DXS 10079	DXS 10074	DXS 10075	Freq.	DXS 7424	DXS 101	Freq
11	17	18	0.0039	13	17	18	0.0039	10	24	0.0078
11	19	16	0.0078	15	14	18	0.0039	11	23	0.0039
11	20	17	0.0039	15	16	16	0.0039	11	24	0.0388
11	21	16	0.0039	15	17	16	0.0039	11	26	0.0116
12	15	16	0.0039	15	17	18	0.0078	11	27	0.0155
12	16	16	0.0039	15	18	17	0.0039	11	28	0.0039
12	16	17	0.0039	15	19	18	0.0039	12	22	0.0155
12	17	15	0.0039	16	15	16	0.0078	12	23	0.0039
12	17	16	0.0039	16	16	16	0.0155	12	25	0.0155
12	18	7	0.0116	16	16	18	0.0078	12	26	0.0078
12	18	17	0.0116	16	17	17	0.0078	12	27	0.0039
12	19	7	0.0078	16	18	17	0.0039	12	28	0.0116
12	19	15	0.0116	17	15	17	0.0078	13	18	0.0039
12	19	16	0.0116	17	16	16	0.0078	13	20	0.0078
12	19	17	0.0039	17	16	17	0.0116	13	22	0.0078
12	19	18	0.0078	17	16	18	0.0039	13	23	0.0155
12	19	19	0.0039	17	17	17	0.0039	13	24	0.0969
12	20	15	0.0116	17	17	18	0.0039	13	25	0.0698
12	20	16	0.0039	17	18	16	0.0078	13	26	0.0271
12	20	17	0.0078	17	18	17	0.0039	13	27	0.0155
12	20	18	0.0116	17	20	16	0.0039	13	28	0.0078
12	21	15	0.0039	18	7	13	0.0116	13	30	0.0039
12	21	16	0.0039	18	15	16	0.0039	14	22	0.0078
12	21	19	0.0039	18	15	18	0.0039	14	23	0.0039
12	22	16	0.0039	18	16	16	0.0194	14	24	0.0426
13	15	14	0.0039	18	16	17	0.0233	14	25	0.0581
13	16	16	0.0116	18	16	18	0.0078	14	26	0.0233
13	17	17	0.0039	18	16	19	0.0039	14	27	0.0194
13	17	20	0.0039	18	17.1	17	0.0039	14	28	0.0194
13	18	15	0.0039	18	17	16	0.0078	14	29	0.0039
13	18	16	0.0233	18	17	17	0.0310	14	31	0.0039
13	18	17	0.0116	18	17	18	0.0078	15	18	0.0039
13	18	17.1	0.0039	18	18	17	0.0078	15	20	0.0039

13	18	18	0.0078	18	18	18	0.0078	15	22	0.0039
13	19	8	0.0039	18	19	19	0.0039	15	23	0.0194
13	19	9	0.0039	19	7	13	0.0078	15	24	0.0543
13	19	15	0.0116	19	8	16	0.0039	15	25	0.0426
13	19	16	0.0116	19	8	17	0.0039	15	26	0.0349
13	19	17	0.0155	19	9	16	0.0039	15	27	0.0271
13	19	18	0.0116	19	9	17	0.0039	15	28	0.0155
13	19	19	0.0078	19	15	16	0.0039	15	30	0.0039
13	20	8	0.0039	19	15	17	0.0194	16	18	0.0039
13	20	16	0.0155	19	15	18	0.0078	16	22	0.0078
13	20	17	0.0194	19	15	19	0.0039	16	24	0.0465
13	20	18	0.0388	19	16	15	0.0078	16	25	0.0349
13	20	19	0.0039	19	16	16	0.0078	16	26	0.0271
13	21	16	0.0039	19	16	17	0.0039	16	27	0.0233
13	21	17	0.0155	19	16	18	0.0155	16	28	0.0194
13	21	18	0.0078	19	16	19	0.0078	16	29	0.0039
13	21	19	0.0039	19	17	16	0.0039	17	15	0.0039
13	21	20	0.0039	19	17	17	0.0233	17	21	0.0078
13	22	16	0.0039	19	17	18	0.0155	17	22	0.0039
13	22	17	0.0078	19	18	16	0.0194	17	24	0.0155
13	22	19	0.0039	19	18	17	0.0271	17	26	0.0039
14	13	17	0.0039	19	18	18	0.0116	18	15	0.0039
14	15	17	0.0116	19	19	17	0.0194	18	25	0.0039
14	15	18	0.0039	19	19	18	0.0116	19	25	0.0039
14	15	19	0.0039	20	7	13	0.0039	10	24	0.0078
14	16	15	0.0039	20	8	18	0.0039	11	23	0.0039
14	16	16	0.0078	20	15.3	18	0.0039	11	24	0.0388
14	16	17	0.0039	20	15	16	0.0039	11	26	0.0116
14	16	18	0.0039	20	15	17	0.0078	11	27	0.0155
14	17	16	0.0078	20	15	18	0.0039	11	28	0.0039
14	17	17	0.0039	20	16	15	0.0039	12	22	0.0155
14	17	18	0.0078	20	16	16	0.0194	12	23	0.0039
14	18	15	0.0039	20	16	17	0.0194	12	25	0.0155
14	18	16	0.0078	20	16	18	0.0155	12	26	0.0078
14	18	17	0.0155	20	16	20	0.0039	12	27	0.0039
14	18	18	0.0039	20	17.3	17	0.0039	12	28	0.0116

14	18	19	0.0039	20	17	15	0.0039	13	18	0.0039
14	19	8	0.0039	20	17	16	0.0116	13	20	0.0078
14	19	9	0.0039	20	17	17	0.0310	13	22	0.0078
14	19	15	0.0039	20	17	18	0.0078	13	23	0.0155
14	19	16	0.0078	20	18	16	0.0194	13	24	0.0969
14	19	17	0.0233	20	18	17	0.0349	13	25	0.0698
14	19	18	0.0388	20	18	18	0.0271	13	26	0.0271
14	19	19	0.0155	20	18	19	0.0039	13	27	0.0155
14	20	7	0.0039	20	19	16	0.0078	13	28	0.0078
14	20	15	0.0039	20	19	17	0.0155	13	30	0.0039
14	20	15.3	0.0039	20	19	18	0.0078	14	22	0.0078
14	20	16	0.0388	20	19	19	0.0078	14	23	0.0039
14	20	17	0.0155	20	20	17	0.0078	14	24	0.0426
14	20	17.3	0.0039	21	15	17	0.0039	14	25	0.0581
14	20	18	0.0271	21	15	18	0.0078	14	26	0.0233
14	20	19	0.0155	21	16	15	0.0039	14	27	0.0194
14	20	20	0.0039	21	16	16	0.0271	14	28	0.0194
14	21	15	0.0039	21	16	17	0.0194	14	29	0.0039
14	21	16	0.0349	21	17	15	0.0039	14	31	0.0039
14	21	17	0.0194	21	17	16	0.0116	15	18	0.0039
14	21	18	0.0116	21	17	17	0.0194	15	20	0.0039
14	21	19	0.0039	21	17	18	0.0078	15	22	0.0039
14	22	16	0.0039	21	18	16	0.0116	15	23	0.0194
14	22	18	0.0194	21	18	17	0.0078	15	24	0.0543
14	22	19	0.0116	21	18	18	0.0078	15	25	0.0426
14	22	21	0.0039	21	19	16	0.0039	15	26	0.0349
15	16	15	0.0039	21	19	17	0.0039	15	27	0.0271
15	17	15	0.0039	21	19	18	0.0078	15	28	0.0155
15	17	16	0.0039	21	20	18	0.0039	15	30	0.0039
15	18	16	0.0155	22	16	16	0.0078	16	18	0.0039
15	18	17	0.0078	22	16	17	0.0039	16	22	0.0078
15	18	18	0.0039	22	17	17	0.0039	16	24	0.0465
15	19	15	0.0039	22	17	18	0.0039	16	25	0.0349
15	19	16	0.0039	22	18	16	0.0078	16	26	0.0271
15	19	19	0.0039	22	18	17	0.0116	16	27	0.0233
15	20	17	0.0078	22	18	18	0.0039	16	28	0.0194

15	20	18	0.0078	22	19	16	0.0039	16	29	0.0039
15	20	19	0.0194	22	19	17	0.0155	17	15	0.0039
15	20	20	0.0039	22	21	17	0.0039	17	21	0.0078
15	21	15	0.0039					17	22	0.0039
15	21	16	0.0039					17	24	0.0155
15	21	17	0.0078					17	26	0.0039
15	21	18	0.0078					18	15	0.0039
15	21	19	0.0039					19	25	0.0039
15	22	19	0.0039							
16	17	16	0.0078							
16	18	16	0.0078							
16	19	15	0.0039							
16	20	16	0.0039							
16	22	18	0.0039							

Supplementary Table S21. Haplotype frequencies observed among the Sri Lankan Tamil male population

DXS 7132	DXS 10079	DXS 10074	Freq.	DXS 10079	DXS 10074	DXS 10075	Freq.	DXS 7424	DXS 101	Freq
12	17	8	0.0130	15	16	16	0.0260	11	22	0.0130
12	18	7	0.0130	16	15	17	0.0260	11	23	0.0390
12	18	18	0.0130	17	8	17	0.0130	11	24	0.0260
12	19	7	0.0130	17	16	17	0.0260	11	25	0.0260
12	19	20	0.0130	17	17	16	0.0260	11	26	0.0390
12	20	15	0.0130	17	18	16	0.0130	11	27	0.0130
12	20	17	0.0130	18	7	13	0.0130	12	26	0.0130
13	15	16	0.0130	18	15	17	0.0130	13	18	0.0130
13	16	15	0.0130	18	16	17	0.0130	13	21	0.0130
13	17	16	0.0130	18	17	17	0.0130	13	23	0.0260
13	17	17	0.0130	18	18	16	0.0130	13	24	0.0909
13	18	18	0.0130	18	18	17	0.0260	13	25	0.0779
13	19	16	0.0260	18	18	18	0.0130	13	26	0.0130
13	19	17	0.0130	18	19	17	0.0130	13	27	0.0260
13	19	18	0.0260	19	7	13	0.0130	13	28	0.0130
13	19	19	0.0130	19	16	16	0.0130	14	21	0.0130
13	20	16	0.0130	19	16	17	0.0519	14	23	0.0260
13	21	7	0.0130	19	16	18	0.0390	14	24	0.0519
13	21	17	0.0130	19	17	16	0.0130	14	25	0.0909
13	21	18	0.0130	19	17	17	0.0390	14	26	0.0260
14	18	15	0.0130	19	17	18	0.0260	14	27	0.0260
14	18	16	0.0130	19	18	17	0.0390	14	28	0.0130
14	18	18	0.0260	19	18	19	0.0130	15	15	0.0130
14	18	19	0.0130	19	19	17	0.0130	15	19	0.0130
14	19	16	0.0260	19	19	18	0.0130	15	23	0.0519
14	19	17	0.0520	19	20	17	0.0130	15	24	0.0390
14	19	18	0.0130	20	15	16	0.0130	15	25	0.0390
14	20	15	0.0130	20	15	17	0.0260	15	26	0.0649
14	20	16	0.0130	20	15	18	0.0130	15	27	0.0260
14	20	17	0.0520	20	16	17	0.0519	15	28	0.0130
14	20	18	0.0260	20	17	17	0.0649	16	15	0.0130
14	20	19	0.0130	20	17	18	0.0130	16	22	0.0130
14	21	15	0.0130	20	18	16	0.0260	16	25	0.0130

14	21	17	0.0260	20	18	17	0.0130	16	28	0.0130
14	21	18	0.0649	20	19	16	0.0130			
14	21	19	0.0130	21	7	14	0.0130			
14	22	16	0.0130	21	15	16	0.0130			
14	22	18	0.0130	21	17	16	0.0390			
14	22	19	0.0130	21	18	16	0.0130			
14	23	16	0.0130	21	18	17	0.0649			
15	15	16	0.0130	21	18	18	0.0260			
15	16	15	0.0130	21	19	16	0.0130			
15	17	16	0.0130	22	16	18	0.0130			
15	17	17	0.0130	22	18	17	0.0130			
15	17	18	0.0130	22	19	16	0.0130			
15	18	17	0.0130	23	16	17	0.0130			
15	19	16	0.0520							
15	19	17	0.0130							
15	20	15	0.0130							
15	20	16	0.0260							
15	20	17	0.0130							
15	20	18	0.0130							
15	21	18	0.0130							
16	19	18	0.0130							
16	19	19	0.0130							
16	20	15	0.0130							
16	21	18	0.0130							

Supplementary Table S22. Haplotype frequencies observed among the Indian Tamil male population

DXS 7132	DXS 10079	DXS 10074	Freq.	DXS 10079	DXS 10074	DXS 10075	Freq.	DXS 7424	DXS 101	Freq
11	19	7	0.0159	15	18	18	0.0159	11	23	0.0159
11	20	17	0.0159	16	18	16	0.0159	11	24	0.0159
12	18	7	0.0318	17	18	16	0.0317	12	24	0.0159
12	18	18	0.0159	18	7	13	0.0635	12	25	0.0159
12	19	17	0.0159	18	15	18	0.0159	13	23	0.0159
12	20	17	0.0159	18	17	17	0.0317	13	24	0.1111
12	20	19	0.0159	18	17	18	0.0159	13	25	0.0317
12	22	19	0.0159	18	18	17	0.0317	13	26	0.0476
12	23	18	0.0159	18	18	18	0.0159	13	28	0.0317
13	17	18	0.0159	18	19	18	0.0159	14	21	0.0159
13	18	7	0.0159	19	7	13	0.0159	14	22	0.0159
13	18	15	0.0159	19	8	15	0.0159	14	23	0.0317
13	18	17	0.0318	19	14	17	0.0159	14	24	0.0476
13	18	18	0.0159	19	16	16	0.0159	14	25	0.0317
13	19	14	0.0159	19	16	17	0.0952	14	26	0.0635
13	19	16	0.0318	19	16	18	0.0159	14	27	0.0159
13	19	17	0.0159	19	17	16	0.0317	15	23	0.0476
13	20	15	0.0159	19	17	17	0.0317	15	24	0.0635
13	20	17	0.0159	19	17	18	0.0159	15	25	0.0794
13	20	18	0.0159	19	18	16	0.0159	15	26	0.0635
13	22	12	0.0159	19	18	17	0.0159	15	27	0.0317
13	22	15	0.0159	19	19	17	0.0159	15	28	0.0159
14	16	18	0.0159	19	20	17	0.0159	16	18	0.0159
14	18	18	0.0159	20	8	15	0.0159	16	23	0.0159
14	18	19	0.0159	20	15	17	0.0159	16	24	0.0317
14	19	16	0.0635	20	16	17	0.0476	16	27	0.0159
14	19	17	0.0159	20	16	18	0.0159	16	28	0.0317
14	19	18	0.0318	20	17	16	0.0159	17	18	0.0159
14	19	19	0.0159	20	17	17	0.0635	17	24	0.0159
14	20	16	0.0476	20	17	18	0.0159	18	19	0.0159
14	20	17	0.0476	20	18	17	0.0317	18	27	0.0159
14	20	18	0.0159	20	18	18	0.0317			
14	20	19	0.0159	20	19	17	0.0317			

14	21	15	0.0159	21	15	16	0.0159
14	21	18	0.0159	21	15	18	0.0159
14	21	19	0.0159	21	18	17	0.0317
15	15	18	0.0159	21	19	17	0.0159
15	17	18	0.0159	22	12	16	0.0159
15	18	7	0.0159	22	15	17	0.0159
15	18	17	0.0159	22	19	17	0.0159
15	19	8	0.0159	23	18	19	0.0159
15	19	16	0.0318				
15	19	20	0.0159				
15	20	8	0.0159				
15	20	16	0.0159				
15	20	18	0.0159				
15	21	15	0.0159				
15	21	18	0.0159				
16	19	17	0.0318				
17	20	18	0.0159				

Supplementary Table S23. Haplotype frequencies observed among the Moor male population

DXS 7132	DXS 10079	DXS 10074	Freq.	DXS 10079	DXS 10074	DXS 10075	Freq.	DXS 7424	DXS 101	Freq
12	18	7	0.0149	16	17	16	0.0149	11	23	0.0149
12	18	15	0.0149	16	18	17	0.0299	11	24	0.0448
12	18	16	0.0149	17	17	18	0.0149	11	25	0.0149
12	18	17	0.0149	17	18	16	0.0149	12	24	0.0299
12	19	15	0.0149	18	7	14	0.0149	12	25	0.0149
12	19	19	0.0149	18	9	14	0.0299	12	26	0.0299
12	20	7	0.0149	18	15	17	0.0299	12	28	0.0149
12	20	17	0.0149	18	16	17	0.0149	12	29	0.0299
12	20	18	0.0299	18	16	18	0.0299	13	23	0.0299
12	21	16	0.0149	18	17	12	0.0149	13	25	0.0597
13	16	18	0.0299	18	17	16	0.0149	13	27	0.0149
13	17	18	0.0149	18	17	18	0.0299	13	29	0.0149
13	18	9	0.0149	18	18	12	0.0149	14	22	0.0149
13	18	16	0.0149	18	18	17	0.0149	14	24	0.0448
13	18	19	0.0149	18	19	17	0.0299	14	25	0.0597
13	19	16	0.0299	19	8	15	0.0149	14	26	0.0299
13	19	17	0.0149	19	15	17	0.0299	14	27	0.0448
13	19	19	0.0149	19	16	15	0.0149	14	28	0.0149
13	20	8	0.0149	19	16	16	0.0149	15	23	0.0597
13	20	17	0.0149	19	16	18	0.0448	15	24	0.0746
13	20	18	0.0597	19	17	15	0.0149	15	25	0.0448
13	21	8	0.0149	19	17	17	0.0299	15	27	0.0448
13	21	16	0.0149	19	17	18	0.0299	15	28	0.0149
13	21	18	0.0149	19	19	17	0.0299	16	18	0.0149
13	22	17	0.0149	19	19	18	0.0149	16	19	0.0149
14	16	17	0.0149	20	7	13	0.0149	16	23	0.0299
14	18	9	0.0149	20	8	17	0.0149	16	24	0.0149
14	18	15	0.0149	20	15	16	0.0149	16	25	0.0448
14	18	17	0.0149	20	16	16	0.0149	16	26	0.0149
14	18	18	0.0149	20	16	18	0.0149	16	28	0.0149
14	18	19	0.0149	20	17	16	0.0149	16	29	0.0149
14	19	15	0.0149	20	17	17	0.0149	17	24	0.0299
14	19	16	0.0149	20	17	18	0.0448	17	26	0.0299

14	19	17	0.0299	20	18	16	0.0448	17	27	0.0149
14	20	16	0.0299	20	18	17	0.0299			
14	20	17	0.0448	20	18	19	0.0149			
14	21	15	0.0149	21	8	19	0.0149			
14	21	18	0.0149	21	15	17	0.0149			
14	21	19	0.0149	21	16	17	0.0149			
14	22	16	0.0149	21	16	18	0.0149			
14	22	17	0.0149	21	17	18	0.0149			
14	22	19	0.0149	21	18	16	0.0149			
15	17	17	0.0149	21	18	17	0.0149			
15	18	16	0.0149	21	19	16	0.0149			
15	18	17	0.0149	21	20	17	0.0149			
15	18	18	0.0149	22	16	17	0.0149			
15	19	8	0.0149	22	17	17	0.0149			
15	19	16	0.0299	22	17	18	0.0149			
15	19	17	0.0299	22	18	17	0.0149			
15	19	19	0.0149	22	19	18	0.0149			
15	21	17	0.0149							
15	21	20	0.0149							
15	22	18	0.0149							
16	18	17	0.0149							
17	20	15	0.0149							

Supplementary Table S24. Compiled data of haplotypes

Cluster I DXS10148-DXS10135-DXS8378

Ethnicity	Sinhalese	SL Tamil	Indian Tamil	Moors
Number of samples	258	77	63	67
Number of observed haplotypes	197	70	56	62
Number of unique haplotypes	151	64	49	58
Percentage of unique haplotypes out total haplotypes	76.65	91.42	87.5	93.55
Freq of most common haplotype	0.01938	0.03896	0.03175	0.04478
Haplotype diversity	0.9987	0.9973	0.9964	0.9973

Cluster II DXS7132-DXS10079-DXS10074-DXS10075

Ethnicity	Sinhalese	SL Tamil	Indian Tamil	Moors
Number of samples	258	77	63	67
Number of observed haplotypes	179	63	56	62
Number of unique haplotypes	129	53	51	58
Percentage of unique haplotypes out total haplotypes	72.07	84.13	91.07	93.55
Freq of most common haplotype	0.023256	0.05195	0.04762	0.04478
Haplotype diversity	0.9962	0.9935	0.9955	0.9973

Cluster III DXS6801-DXS6809-DXS6789

Ethnicity	Sinhalese	SL Tamil	Indian Tamil	Moors
Number of samples	258	77	63	67
Number of observed haplotypes	113	57	46	48
Number of unique haplotypes	61	42	34	36
Percentage of unique haplotypes out total haplotypes	53.98	73.68	73.91	75
Freq of most common haplotype	0.03876	0.05195	0.0635	0.0597
Haplotype diversity	0.9879	0.9908	0.9882	0.9873

Cluster IV DXS7424-DXS101-DXS7133

Ethnicity	Sinhalese	SL Tamil	Indian Tamil	Moors
Number of samples	258	77	63	67
Number of observed haplotypes	115	56	50	51
Number of unique haplotypes	59	43	39	39
Percentage of unique haplotypes out total haplotypes	51.3	76.79	78	76.47
Freq of most common haplotype	0.05039	0.06494	0.04762	0.05970
Haplotype diversity	0.9882	0.9887	0.9923	0.9905
